# The Potential Role of Direct and Indirect Contacts on Infection Spread in Dairy Farm Networks

**DOI:** 10.1371/journal.pcbi.1005301

**Published:** 2017-01-26

**Authors:** Gianluigi Rossi, Giulio A. De Leo, Stefano Pongolini, Silvano Natalini, Luca Zarenghi, Matteo Ricchi, Luca Bolzoni

**Affiliations:** 1 Dipartimento di Bioscienze, Università degli studi di Parma, Parco Area delle Scienze, Parma, Italy; 2 Risk Analysis Unit, Istituto Zooprofilattico Sperimentale della Lombardia e dell’Emilia Romagna, Via dei Mercati, Parma, Italy; 3 Stanford University, Hopkins Marine Station, Pacific Grove, CA, United States of America; 4 Servizio Veterinario e Igiene Alimenti, Assessorato Politiche per la Salute Regione Emilia-Romagna, Viale Aldo Moro, Bologna, Italy; 5 Servizio Igiene degli Allevamenti e Produzioni Zootecniche, AUSL di Parma, Via Vasari, Parma, Italy; 6 National Reference Centre for Paratuberculosis, Istituto Zooprofilattico Sperimentale della Lombardia e dell'Emilia Romagna, Strada Faggiola 1, loc. Gariga—Podenzano (PC), Italy; The Pennsylvania State University, UNITED STATES

## Abstract

Animals’ exchanges are considered the most effective route of between-farm infectious disease transmission. However, despite being often overlooked, the infection spread due to contaminated equipment, vehicles, or personnel proved to be important for several livestock epidemics. This study investigated the role of indirect contacts in a potential infection spread in the dairy farm network of the Province of Parma (Northern Italy). We built between-farm contact networks using data on cattle exchange (direct contacts), and on-farm visits by veterinarians (indirect contacts). We compared the features of the contact structures by using measures on static and temporal networks. We assessed the disease spreading potential of the direct and indirect network structures in the farm system by using data on the infection state of farms by paratuberculosis. Direct and indirect networks showed non-trivial differences with respect to connectivity, contact distribution, and super-spreaders identification. Furthermore, our analyses on paratuberculosis data suggested that the contributions of direct and indirect contacts on diseases spread are apparent at different spatial scales. Our results highlighted the potential role of indirect contacts in between-farm disease spread and underlined the need for a deeper understanding of these contacts to develop better strategies for prevention of livestock epidemics.

## Author Summary

Farm-to-farm contacts due to shared operators and vehicles–such as veterinarians, hoof-trimmers, milk and rendering trucks–are generally considered important for the spread of many infectious diseases in livestock systems. These contacts are usually defined as *indirect*, as opposed to animal movements which are defined as *direct contacts*. The actual significance of *indirect contacts* is still poorly understood due to study limitations deriving from their highly diverse and complex nature and to privacy issues in data collection. Thanks to the availability of high-resolution data in space and time on veterinarian on-farm visits in a dairy farm network in Northern Italy, we showed through network analysis techniques that between-farm *indirect contacts* are more widespread and display significantly different patterns compared to *direct contacts*. Using infection data on paratuberculosis, we also found that indirect contacts can support disease spread at local scale, while direct contacts (due to long distance animals exchanges) can support disease spread on a larger spatial scale. Our results corroborate the conclusion that the role of indirect contacts in livestock disease spread is essential and deserves a deeper understanding.

## Introduction

The structure of contact networks between individuals from human and animal populations is a key determinant of the dynamics of communicable diseases. In response to the Bovine Spongiform Encephalopathy crisis, the Council of the European Union implemented in 1997 a system of permanent identification of individual bovine animals enabling reliable traceability from birth to death. A fundamental part of this system consists in an extensive database to track movements of farmed animals. Information on between-farm animal movements have been used to reveal the existing contact network structure within livestock systems [1–5], as it is considered the most effective route of disease transmission [6]. The study of the disease spread in livestock systems made it possible to fine tune surveillance systems, to address biosafety guidelines and control strategies aimed to reduce the risk of disease outbreaks, and to limit the impact on animal health and economic sustainability of farming systems.

The vast majority of these studies, models and analyses mainly (if not exclusively) focuses on direct contacts, i.e. infections following the movement of diseased animals between farms. Alternative and often more cryptic transmission pathways have received much less attention, even though they can crucially affect the efficacy of disease surveillance and control systems. For instance, in 2001 foot-and-mouth Disease (FMD) epidemic in the UK, although animal movements were banned since late February, the on-set of newly infected farms was reported until the end of the summer [7,8]. Since further studies excluded a strong effect of aerial spread for the FMD virus strain in question, the only alternative route of transmission for the infection was represented by the spread through fomites [9]–such as contaminated operators, vehicles, and equipment–potentially capable of carrying pathogens from infected farms to susceptible ones [10]. In fact, the FMD epidemic stopped only after the implementation of stronger biosecurity measures in UK farms, which mostly targeted the movement of contaminated equipment and personnel [8].

Between-farm disease transmission through fomites, mediated by operators and personnel external to the farm, is usually defined as indirect transmission [11]. In recognition of its importance, epidemiological models of disease dynamics in livestock have started to include different pathways of between-farm transmission in addition to cattle movements (see [12], and references therein).

Unlike animal movements, the role of indirect transmission of livestock diseases is still largely unknown. The reason for this knowledge gap is twofold: on the one hand, because of the subtle, highly diverse and complex nature of indirect contacts, it is intrinsically difficult to assess the relative importance of alternative transmission pathways for disease risk. On the other hand, because of privacy reasons, it is much easier to track livestock movements than that of farm operators and personnel. Indeed, collecting reliable and extensive quantitative data on indirect contacts on a temporal and spatial scale relevant for epidemiological modelling has proved to be very challenging. Information on farm operator movements has been generally gathered by using voluntary questionnaires on the number and the frequency of farm visits over a given time span [10,11,13–15]. Despite the usually low questionnaire response rate and the low number of farms involved in these studies, this approach has been crucial to provide a preliminary rank of categories of indirect contacts by potential disease risk. However, the information gathered has been often insufficient to fully investigate the network structure of indirect contacts in a given area, and to characterize the contact frequency between farms. This is why only few studies have applied network analysis techniques on questionnaire-based data (see e.g. [10,16,17]).

Alternatively, indirect routes of transmission in epidemic models have been represented using risk kernel functions [18,19]. These are functions assigning a probability of between-farm disease transmission on the basis of the inter-farm distance [19,20]. However, the approach based on kernel functions is unable to tease apart the relative importance of different networks of indirect contacts, such as those associated to the movement of different farm operators.

The aim of this work is to present a novel quantitative analysis of the relative importance of indirect and direct contacts in a network of 1,349 dairy farms in the Province of Parma in the Emilia Romagna Region (Italy). The analysis is based on a unique, high-resolution temporal and spatial database of between-farm movements of 50 public officers of the regional veterinary service and 203 private veterinary practitioners (which represent the potentially infectious indirect contacts). The former visit a large number of farms usually only few times a year; the latter serve a small subset of farms each, and they visit them several times per year. We thus expected that the structures of their contact networks are substantially different and might result in different risks of disease transmission through fomites. We used network analysis to characterize the structure of the networks of indirect contacts and contrast them with the structure of the network of direct contacts (i.e. the one associated to animal movement/trade). Then, we assessed the contribution of both direct and indirect contacts networks in explaining the observed spatial distribution of dairy farms infected by *Mycobacterium avium* subsp. *paratuberculosis* (MAP). MAP is responsible for Johne’s disease, a chronic gastrointestinal inflammation affecting ruminants and it is endemic in the study area [21]. It is well documented that animal movements represent the primary route of MAP transmission between farms [22,23]. However, the role of fomites such as footwear [24] and shared farm and veterinary equipment [25] as secondary transmission routes has been highlighted. Finally, we used advanced techniques in network analysis to characterize the temporal network defined by direct and indirect contacts in order to understand the between-farm transmission for fast spreading diseases where the time scale of epidemics is similar to those of the evolution of the network, such as FMD [26].

## Materials and Methods

### Dairy farms and cattle movement data

**Fig 1:**
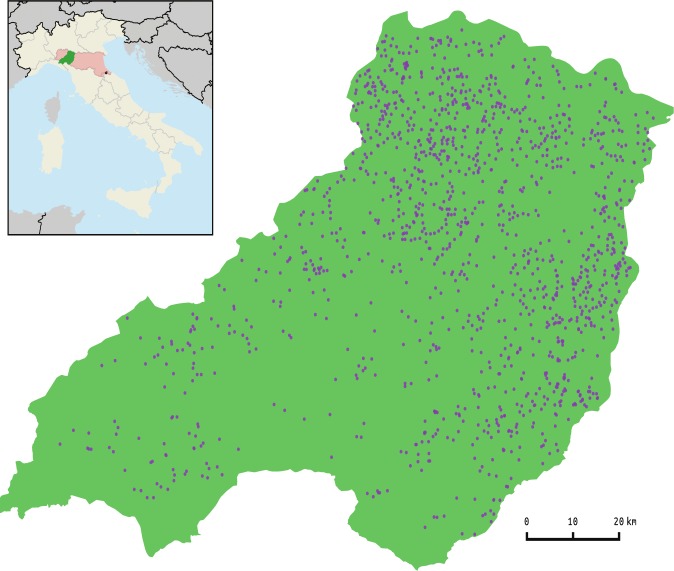
Parma Province dairy system. The Province of Parma (green) and Emilia-Romagna region (red). Blue dots correspond to the dairy farms active in 2013.

Our study system is represented by a network of 1,349 dairy farms operating in the Province of Parma (Emilia-Romagna region, Italy) in 2013 (Fig 1). For each farm, we extracted from the Italian National Bovine Database (BDN) a unique identification code and the related spatial coordinates. As we were interested in analysing the structure of the cattle movement network on a wider geographical scale and time window as well, we extracted from BDN also information on cattle movement from the 4564 dairy farms operating in the whole Emilia-Romagna region (which includes also the province of Parma) between 2010–2013. Each individual cattle movement record contained: a unique identification code for the animal, identifier codes of the farms of origin and destination, codes for farm production sector (dairy or mixed), and the movement date.

As the Province of Parma is a strongly oriented dairy area, beef farms were not considered in the present study. In fact, they represented less than 25% of the total cattle farms area (473 over 1,822), and the two systems are almost completely separated. The only unidirectional contact points consist in the shipment of surplus individuals from dairy, mostly male calves, to beef farms. Beef farms are less involved in veterinarians networks too. First, they do not receive frequent inspections because they are not included in surveillance plans for most diseases (i.e. bovine tuberculosis [27]). Second, beef animals receive less care by practitioners, in part because individuals’ life span is shorter (2 vs. 5 years for dairies [27]), but also because the lower economic value of individuals does not justify intensive health assistance as for dairy cattle.

The network of cattle movements was assembled by creating a directed edge between any two farms (representing network nodes) that exchanged animals during the observed period and setting a non-zero value in the corresponding adjacency matrix [28]. Among the many ways in which edges could be weighted in a time-aggregated cattle movement network [29], we considered (*i*) the unweighted case, in which a link value is equal to 1 if at least one contact exists in a given period, and 0 otherwise; and (*ii*) the case in which links are weighted proportionally to the number of animals exchanged through each contact within a given period.

### Veterinarians movement data

Data on visits of veterinary officers (VO) were provided by the Local Health Unit of Parma Province (LHU). These visits were scheduled by the LHU for various purposes, including animal health inspection and disease surveillance. We created a database including all visits on dairy farms during the year 2013. For each visit we recorded the farm unique identifier, the identifier of the VO visiting the farm (in an anonymous form), and the visit date.

Data on veterinary practitioners (VP) were obtained from three datasets. The first was the list of drug prescriptions in 2013, that is, documents that compulsorily need to be (*i*) signed by a registered practitioner, and (*ii*) delivered and kept by both the LHU and the farm. For this reason, each drug prescription corresponds to at least one on-farm visit by a veterinarian. This dataset contained the unique identifier of the farm where the drug was prescribed, the identifier code of the VP prescribing the drug (in an anonymous form), and the prescription date. The second dataset was constituted by the records of diagnostic samples submitted to the Istituto Zooprofilattico Sperimentale della Lombardia e dell’Emilia-Romagna (IZSLER), the local veterinary diagnostic laboratory. These samples are collected on farm by practitioners and delivered to the IZSLER for biological testing. This dataset contained the unique identifier of the farm where the sample was collected, the identifier of the VP collecting the sample (in an anonymous form), and the date of collection. The third dataset included the list of on-farm inspections on order of the LHU, but subcontracted to VPs. For each dataset, the record contained the VP identifier, the farm unique identifier, and the visit date.

To build the VO and the VP contact networks, we assigned a directed edge connecting a given farm to those later visited by the same veterinarian within a given time interval. This time interval represents the time span in which the veterinarian or her/his equipment can remain contaminated by the pathogenic agent. We defined it as contamination period, *h*, and it depends on the pathogen ability to survive in the environment and on the type of contaminated material.

The order by which VO and VP operators visited the farms within a given day was not reported in the dataset. Thus, to define the contact chain generated by multiple farm visits occurring in any given day, we generated 50 networks with potential itineraries by randomly selecting, for each veterinarian, the first farm visited in that day, so to derive the itinerary that minimized travel distance (see S1 Text for details on method and software).

The analyses of network structure and disease risk were conducted by setting *h* = 0 days under the conservative assumption that only within day visits can result in infection transmission. We assessed the effect on network properties of higher values for *h* in a specific analysis reported in S1 Text. As for the cattle movement network, we also developed a weighted version of the veterinarian networks, where the weighting coefficients represent the number of contacts within the year 2013.

### Structural properties of networks

To evaluate the potential risk of disease spread in the three different networks (CM, VO, and VP), as per other studies on farm networks [2,3,30], we first derived three important metrics for each network, namely: (*i*) the link density [31], which is defined as the fraction of observed links over the possible number of links; (*ii*) the giant strongly connected component (GSCC) [2], which is defined as the biggest portion of the network in which each node is reachable from any other node; and, in the weighted version of the networks, (*iii*) the contact frequency, which is defined as the mean frequency of the observed links within the observation period.

The node degree *k*, a central measure in network theory, is defined as the number of links for each node [28]. As our networks were characterised by directed links, we derived two node degrees for each farm in each contact network: the *i*-th farm in-degree, defined as the total number of farms from which farm *i* receives cattle or farms visited by VO and VP before visiting farms *i* (*k*_*I*_); and *i*-th farm out-degree, defined as the number of farms to which farm *i* sends cattle or farms visited by VO and VP after visiting farm *i* (*k*_*O*_). Consequently, for each contact network, we computed the degree distributions *P*(*k*_*I*_) and *P*(*k*_*O*_), respectively. In the case of VO and VP networks, we computed degrees and degree distributions assuming *h* = 0 days (analyses based on different assumptions on *h* are shown in S1 Text). In addition, in the case of weighted networks, we computed the node strengths, which are defined as the sum of all incoming (in-strength, *S*_*I*_) and outgoing (out-strength, *S*_*O*_) links' weights [28]. As for the degrees, for each weighted contact network, we computed the strength distributions *P*(*S*_*I*_) and *P*(*S*_*O*_) (see S1 Text for details).

### Define farm exposure to infection

A fundamental assumption in the spatial analysis of epidemiological data for communicable diseases is that the contact network is an important driver of the observed disease dynamics and, accordingly, may be able to partially explain the spatial distribution of reported cases of the infectious disease under study. Here, we wanted to assess the relationship between direct and indirect contact networks and *Mycobacterium avium* subsp. *paratuberculosis* (MAP) positive farms at regional scale (i.e. Emilia-Romagna, for cattle movements only) and local scale (i.e. Parma province, for veterinarians and cattle movements) by using data on the infection status of 2,648 dairy farms in Emilia-Romagna region (whereof 966 in Parma province) as identified in Ricchi et al. [21]. The infection state of farms, positive or negative, was evaluated by ascertaining the presence of MAP in bulk tank milk by real-time PCR targeting insertion sequence IS900, twice per farm [21]. To avoid detection bias, farms for which bulk tank milk had not been tested for MAP were excluded from the analysis.

Since MAP has a long incubation period [32] and, consequently, a slow infection dynamics, static network measures as those underlying our analysis can be appropriately used to represent the between-farm disease transmission, as shown by [26]. To assess the relationship between MAP presence and direct/indirect contact structures, we used a network-based model approach similar to that developed in [16]. Specifically, we defined the mean exposure to infection of MAP positive farms (*E*_*I*_) as:

$$E_{I}=\frac{\sum_{j=1}^{n}\sum_{i\neq j}E_{ij}\delta_{j}}{\sum_{j=1}^{n}\delta_{j}},$$ (1)

where *δ*_*j*_ = 1 [*δ*_*j*_ = 0] if farm *j* is MAP positive [negative]; and *E*_*ij*_ represents exposure of farm *j* to farm *i*, defined as *E*_*ij*_ = *A*_*ij*_ (where ***A*** represents the adjacency matrix of the weighted network) if farm *i* is MAP positive; *E*_*ij*_ = 0 otherwise. Consequently, ∑_*i* ≠ *j*_*E*_*ij*_ represents the total exposure for farm *j*. Analogously, we defined the mean exposure to infection of MAP negative farms (*E*_*S*_) as:

$$E_{S}=\frac{\sum_{j=1}^{n}\sum_{i\neq j}E_{ij}\theta_{j}}{\sum_{j=1}^{n}\theta_{j}},$$ (2)

where *θ*_*j*_ = 1 [*θ*_*j*_ = 0] if farm *j* is MAP negative [positive]. Garcia Alvarez et al. [16] proposed that, if the infection was transmitted from the observed contact networks, we would expect that MAP positive farms were more exposed than MAP negative ones (i.e., *E*_*I*_ > *E*_*S*_). In addition, we would expect that MAP positive farms were more exposed in the observed contact network than in a random one, while MAP negative farms were expected to be less or similarly exposed compared to a random network. To test these hypotheses, we generated random networks with the same number of nodes as in the observed contact networks, but randomly allocating the edges between the nodes. In order to investigate the impact of the network linking pattern (instead of the in-degree distribution) on the spread of the disease, the distribution of farm contacts was maintained through a rewiring process in the random networks, as suggested by Kiss et al. [33]. The node state with respect to MAP infection was maintained fixed in all random networks using the observed bulk tank milk data.

Since MAP can persist in farms for years, we also tested whether our results were robust with respect to the specific year used to derive the contact networks. In particular, for the cattle movement network, for which data were available for multiple years, we tested whether contact networks built by using data from years 2010–2012 could explain the infectious state by MAP detected in 2013. In addition, since in the absence of biosecurity measures MAP can persist in the environment, we tested whether our results at local and regional scale were robust with respect to different assumptions on the contamination period (specifically, *h* = 0 and *h* = 7 days).

Moreover, to determine the possible effect of spatial clustering as a driver of the differences in MAP positivity among farms, we used the *q*-nearest neighbours test. The *q*-nearest neighbours is a non-parametric test able to identify a potential spatial clustering in the distribution of cases, by computing the number of cases observed within the *q* neighbour farms of each positive case [39]. We set the number of neighbours *q* from 1 to 10 [16,34].

### Define infection potential and super-spreaders

A major focus of our work was to identify which farms could act as super-spreaders in the studied networks. A super-spreader is defined as a highly connected individual farm able to potentially spread the infection to a very large number of farms in the system [35]. In the context of diseases spreading through a contact network, centrality measures are often used to identify the super-spreaders [36]. The most simple but still effective centrality measure is the degree centrality [28]. However, despite the simplicity and the usefulness of the degree centrality, it is well known that assuming a static network derived by all the contacts that occurred in a given period, 2013 in our case, and ignoring the temporal sequence of connections, can lead to largely overestimate the transmission risk for fast spreading diseases, such as FMD and influenza, where the time-scale of the epidemics is similar to that of the evolution of the network [4,26,37,38]. To overcome this limit, Dubé and colleagues [3] introduced a risk-based measure that accounts for the temporal sequence of contacts in animal trade networks, called the infection chains (*IC*). In particular, for a given farm, the ingoing *IC* (*IIC*) and the outgoing *IC* (*OIC*) measure the maximum number of farms connected with it through a sequence of animal movements [3,30]. Konschake et al. [39] extended the infection chain concept by assuming that only contacts occurring within a finite infectious period (γ) may act as potential transmission events. Accordingly, we computed time-dependent *ICs* referred to a specified date of emergence of the infection in the farm system (*d*), specifically *IIC*(*d*) and *OIC*(*d*). From an epidemiological view point, the *OIC*(*d*) in the *i*-th farm, *OIC*_*i*_(*d*), provides an upper bound to the size of an epidemic emerging from the *i*-th farm in day *d*. Analogously, the *IIC*(*d*) in the *i*-th farm, *IIC*_*i*_(*d*), provides an upper bound for the probability for the *i*-th farm of getting infected on day *d* following an epidemic event occurred in any other farm in the network. This was computed as *IIC*_*i*_(*d*)/(*N*−1), with *N* corresponding to the total number of farms in the system. Following these considerations, we defined the *infection potential ρ*_*i*_(*d*) of the *i*-th farm in a given day *d* as:

$$\rho_{i}(d)=\frac{{\text{IIC}}_{i}(d)}{N-1}{\text{OIC}}_{i}(d).$$ (3)

From expression (3), we built two more general epidemiological indicators: the mean *infection potential* of *i*-th farm on a given time-period of *m* days as:

$$\rho_{i}=\frac{\sum_{\text{d}=1}^{m}\rho_{i}(d)}{m}$$ (4)

and the average *infection potential* of the system in day *d* as:

$$\rho (d)=\frac{\sum_{\text{i}=1}^{N}\rho_{i}(d)}{N}.$$ (5)

In order to avoid boundary effects due to the limited period of data availability (1 year), we computed *ρ*_*i*_(*d*) for a period of 245 days starting from the beginning of March to the end of October 2013.

We computed the *infection potential* for individual CM, VO and VP networks, for the veterinarians total network (VT = VO + VP networks), and for the network of all transmission routes combined (TN = CM + VT). For VO, VP, VT, and TN networks, the calculation was repeated for each of the 50 simulations. We defined as super-spreaders the farms in the highest 5^th^ percentile of *ρ*_*i*_ value distribution. We assumed a farm infectious period length γ of 14 days. According to Konschake et al. [39], this is the threshold value above which the *IC* measure is stable to variations in γ and, additionally, this infectious period is compatible with rapidly spreading diseases, such as FMD [40]. However, we performed a sensitivity analysis on the *ICs* for different values of γ (from 3 to 28 days) to assess whether a variation of γ has strong consequences on farm ranking in terms of γ and, thus, on the identification of super-spreaders. In order to assess the correlation between *ρ*_*i*_ calculated for CM and VT networks, we used the Kendall's *τ*, a non-parametric test.

## Results

### Cattle movement network

There were 16,647 cattle moved in the province of Parma in 2013, for a total of 1,433 between-farm directed links in the yearly aggregated CM network. Link-density was 0.0008 (see Table 1). The giant strongly connected component (GSCC) included 18 farms, corresponding to the 1.14% of the network, while the distribution of the yearly exchanged animal-per-contact was very heterogeneous, with an average of 11.62 animals.

**Table 1:** Networks structure measures. Network measures (link density, GSCC, and contacts frequency) for cattle movement (CM) network, veterinary officers (VO) network (median, 5^th^ and 95^th^ percentile over 50 simulations), and veterinary practitioners (VP) network (median, 5^th^ and 95^th^ percentile over 50 simulations).

Network		CM	VO	VP
***Link density***	Median	0.0008	0.0049	0.0029
	*5*^*th*^ *percentile*	*-*	*0*.*00476*	*0*.*00285*
	*95*^*th*^ *percentile*	*-*	*0*.*00501*	*0*.*00292*
***GSCC (% on the network)***	Median	1.33	68.12	54.00
	*5*^*th*^ *percentile*	*-*	*34*.*54*	*53*.*30*
	*95*^*th*^ *percentile*	*-*	*71*.*35*	*54*.*97*
***Average contacts frequency***	Median	11.62	1.18	1.19
	*5*^*th*^ *percentile*	*-*	*1*.*15*	*1*.*18*
	*95*^*th*^ *percentile*	*-*	*1*.*22*	*1*.*20*

**Fig 2:**
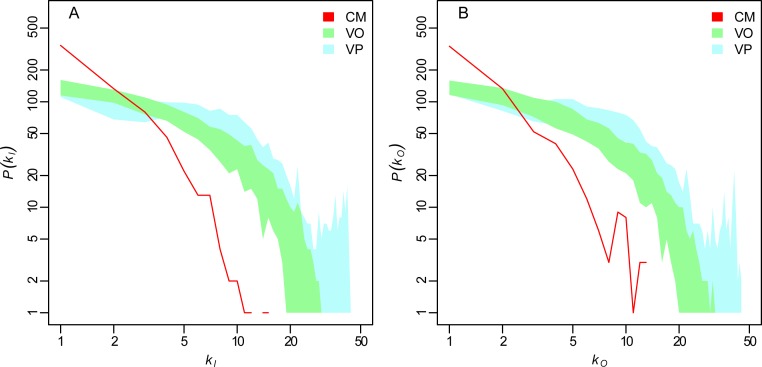
Farm degree distributions. Farm degree distributions (in a log-log scale) of: cattle movement (CM) network (observed, red line); veterinary officer network (VO, 50 simulations, green shade); veterinary practitioner network (VP, 50 simulations, blue shade). Panels a) and b) correspond to *k*_*I*_ (in-degree) and *k*_*O*_ (out-degree), respectively.

The mean farm degree was 1.06, and both *k*_*I*_ and *k*_*O*_ ranged from 0 to 15. The degree distributions *P*(*k*_*I*_) and *P*(*k*_*O*_) are showed in Fig 2 (red line, *a* and *b* panels, respectively). The median of both *k*_*I*_ and *k*_*O*_ was equal to zero, and this was a consequence of the large number of farms with no incoming (688) or outgoing (719) cattle movements within the system during 2013.

The total number of moved cattle between dairy farms within the Emilia-Romagna region ranged from 50,186 to 57,276 over the period 2010–2013. These movements originated from 4,624 to 5,094 between-farm contacts.

### Veterinary networks

The veterinary officer (VO) dataset included data on 6,524 on-farm visits performed by 50 officers. By setting the contamination period *h* equal to 0 (corresponding to assuming possible indirect transmission only for consecutive visits on the same day), the median [5^th^-95^th^ percentile among the 50 simulations] link density was 0.0049 [0.0047–0.0050]; the median [5^th^-95^th^ percentile] giant strongly connected component (GSCC) included about 67% [35%-70%] of the network, corresponding to 918 [480–948] farms. The median [5^th^-95^th^ percentile] yearly contact frequency was 1.19 [1.15–1.22].

The overall veterinary practitioner (VP) dataset included a total of 14,053 visits performed by 203 practitioners. This was the result of joining three data sources: the drug prescription list (11,611 prescriptions by 181 VP), the animal tissue-drop records (1,085 records by 108 VP), and the government subcontracted visits (1,426 visits performed by 12 VP). As for the VO network, we simulated the same day visit order 50 times. The median [5^th^-95^th^ percentile] link density was 0.0029 [0.0029–0.0029]; the median [5^th^-95^th^ percentile] GSCC included about 54% [53%-55%] of the networks, corresponding to 732 [721–740] farms; and the median [5^th^-95^th^ percentile] yearly contact frequency was 1.19 [1.18–1.20]. VO and VP networks showed more widespread degree distributions with respect to the CM network (Fig 2). At *h* = 0, the mean degrees were 6.55 and 3.90 for VO and VP, respectively, and 1.06 for CM. On the other hand, the strength distributions of all three networks showed to be more similarly distributed (see Figure S1.3.1 and Table S1.2.2 in S1 Text).

### Farm exposure to infection

**Fig 3:**
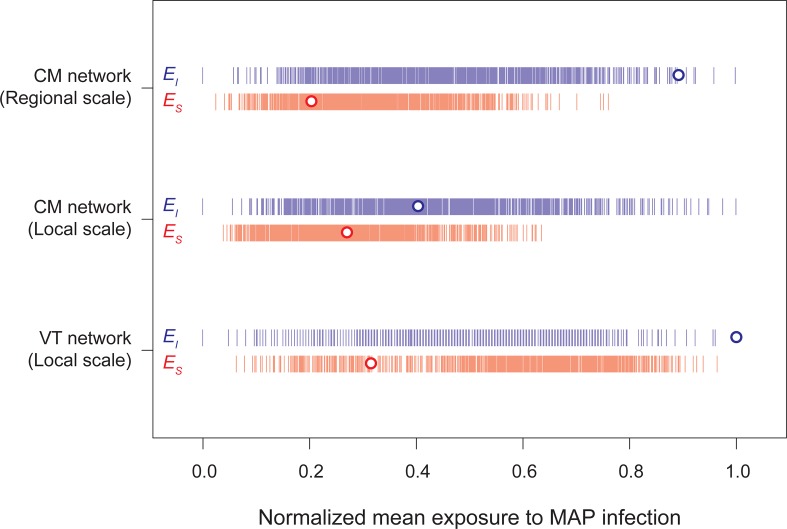
Farm exposure to MAP infection. Mean exposure to MAP-positive (blue dots, *E*_*I*_) and MAP-negative (red dots, *E*_*S*_) farms normalized between zero and one in: upper) cattle movements network (CM) of Emilia-Romagna region (i.e. regional scale); middle) cattle movement network of Parma Province (i.e. local scale); and bottom) the veterinary network (VT) of Parma Province. Vertical bars represent the mean exposure to MAP in random networks with the same distribution of farm contacts as in the observed networks.

As expected from previous literature on between-farm transmission of *Mycobacterium avium* subsp. *paratuberculosis* (MAP), we found evidence of association between cattle movements and the distribution of MAP positive farms within the Emilia-Romagna region (see Fig 3). Specifically, we found that the mean exposure of MAP positive farms (Fig 3 upper segment: blue dot, *E*_*I*_) derived by using the CM network at regional scale is: a) higher than that of MAP negative farms (Fig 3 upper segment: red dot, *E*_*S*_); b) higher than that in a randomly generated network (Fig 3 upper segment: blue vertical bars, *p* = 0.005). Conversely, within Parma province, we did not find a significantly higher mean exposure of MAP positive farms derived by using the CM network (Fig 3 middle segment: blue dot) compared to random networks (Fig 3 middle segment: blue vertical bars, *p* = 0.456). On the other hand, within Parma province, we found that the mean exposure of MAP positive farms in the veterinary network (Fig 3 bottom segment: blue dot) was significantly higher than in random networks (Fig 3 bottom segment: blue vertical bars, *p* < 0.001). In addition, we found that our results were robust with respect to the year considered for building the contact networks and the assumptions on the length of the contamination period (see S1 Text).

**Fig 4:**
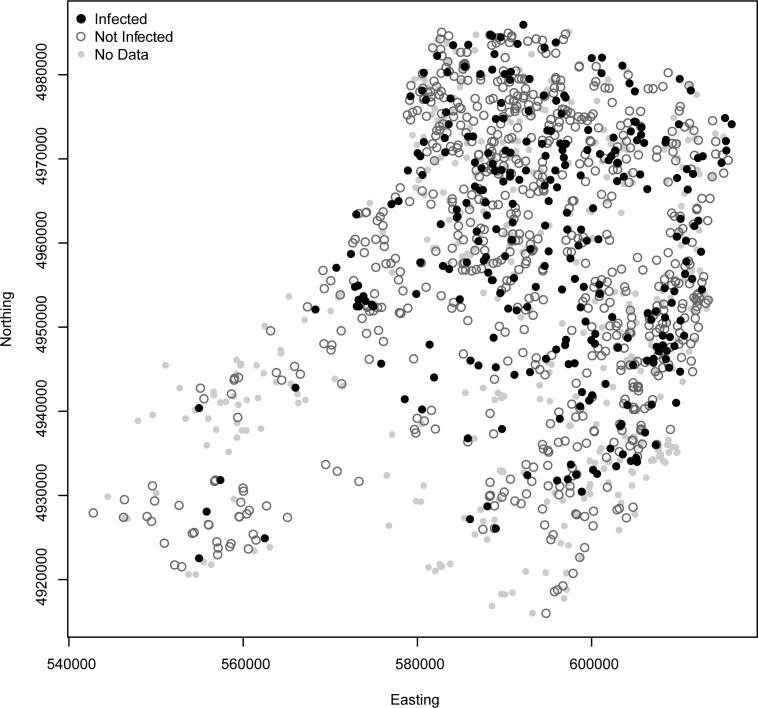
MAP spatial distribution. Spatial distribution of the infection state of farms by *Mycobacterium avium* subsp. *paratuberculosis* (MAP) in the Province of Parma. Black dots correspond to infected farms; dark-grey circles to non-infected farms; and small light-grey dots to no-data farms.

The spatial analysis showed that no clear spatial clustering could be detected among MAP-positive farms. Specifically, the *q*-nearest neighbours test was not statistically significant for neighbour farms from *q* = 1 to *q* = 10 (see Table 2). This result suggests that between-farm transmission of MAP was not associated to spatial proximity (see Fig 4).

**Table 2:** *q*-nearest neighbours test. Results of the *q*-nearest neighbours test on the infection state of farms by *Mycobacterium avium* subsp. *paratuberculosis* (MAP) within the dairy farms of the Province of Parma. Columns show the number *q*-nearest neighbours selected for the analysis (i.e. total number of cases within the number of *q* neighbours of each farm within the system) and corresponding p-value (*p*).

#q	Total *q*-nn	*p*
1	84	0.328
2	166	0.311
3	255	0.164
4	332	0.245
5	426	0.101
6	497	0.216
7	579	0.208
8	664	0.168
9	755	0.101
10	844	0.064

### Infection potential and super-spreaders detection

**Fig 5:**
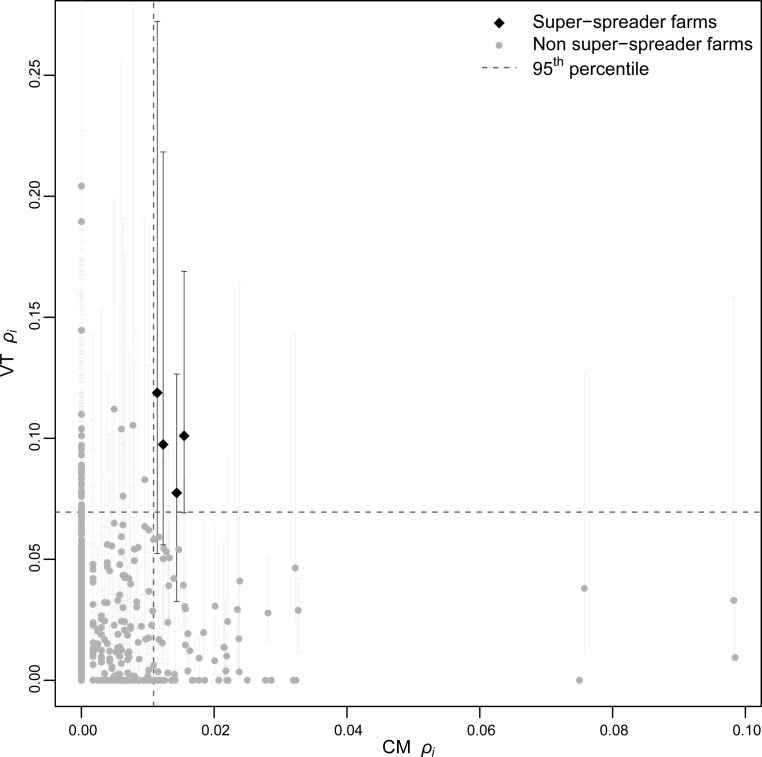
Super-spreaders sharing. Farm *infection potential ρ*_*i*_ (squared values) computed for cattle movement (CM) network (*x-axis*) vs. veterinarians total (VT) network (*y-axis*, median of 50 simulations). Black diamonds correspond to the super-spreader farms; grey dots to the non super-spreaders (vertical bars show the variability over 50 simulations). Dashed lines show the 95^th^ percentile for both measures.

*Infection potential ρ*_*i*_ of VT network was poorly correlated with that of CM network (Kendall's *τ* = 0.08, *p* < 0.01). The number of shared super-spreaders for the CM and VT networks (defined as the 5% of farms with the highest *ρ*_*i*_ value) ranged between 4 and 7 over 50 simulations of within day veterinary itineraries. By using the median value of *ρ*_*i*_ for each farm as a reference measure, the number of shared super-spreaders between direct and indirect contacts was 4 (see Fig 5). To test whether the number of observed shared super-spreaders was significantly higher than in the random case, we computed a permutation test by assigning the observed *ρ*_*i*_ values in each network to random farms. Upon 20,000 runs (1,000 for each of the 50 simulations), the test turned out to be not statistically significant (*p* = 0.20).

**Fig 6:**
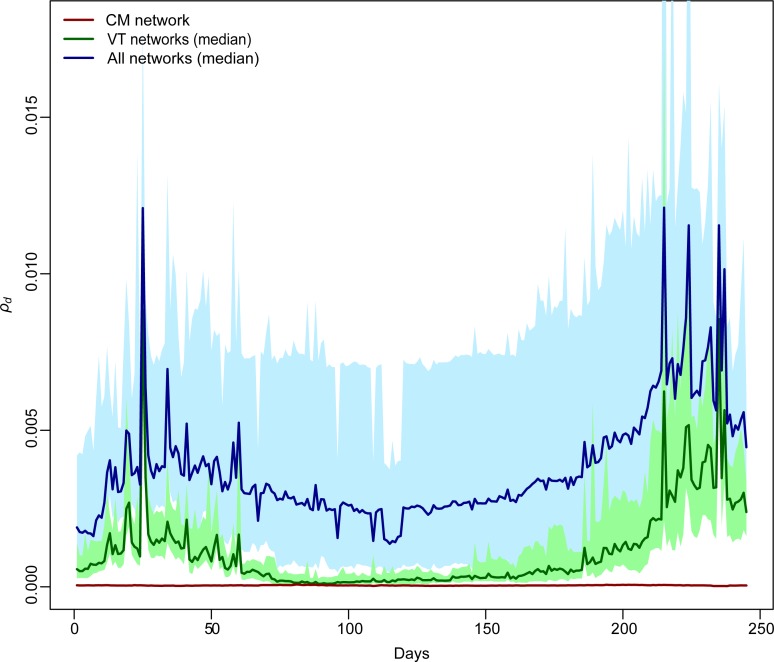
Transmission routes *infection potential*. Daily system *infection potential ρ*(*d*) for cattle movement (CM) network (red line), the veterinarians total (VT) network (green shade results for 50 simulations, median case dark green line), and all networks combined (TN, blue shade results for 50 simulations, median case dark blue line).

Fig 6 shows the time trend of the average *infection potential* of the whole farm system, *ρ*(*d*), during the 245-day period. The average *infection potential ρ*(*d*) was: 0.04 × 10^−3^ (*sd* = 0.44 × 10^−3^) for the CM network, 0.14 × 10^−3^ (*sd* = 0.37 × 10^−3^) for the VO network, and 0.16 × 10^−3^ (*sd* = 0.69 × 10^−3^) for the VP network. Combining networks together, the average *ρ*(*d*) was 0.84 × 10^−3^ (*sd* = 2.17 × 10^−3^) for the veterinarians total network (VT), while *ρ*(*d*) was 3.13 × 10^−3^ (*sd* = 39.23 × 10^−3^) for all the direct and indirect contact networks combined. Sensitivity analyses showed stable rankings for *IIC*, *OIC* and *ρ* with varying infectious period γ (see S1 Text).

## Discussion

### On static and temporal networks

Prevention measures such as targeted surveillance and farm isolation, which are largely used to control livestock epidemics, have been shown to be more effective when directed to those higher risk farms [1,4], also called super-spreaders. Understanding the structure of the contact network is therefore crucial to derive effective surveillance strategies. Identification of super-spreaders, however, should be performed by accounting for all transmission pathways and not only the direct ones.

In fact, despite it is well known that indirect contacts are less efficient in transmitting infectious diseases compared to direct ones [6,40], our results showed that they can substantially affect the ability of farms to potentially spread a disease within the network system. In particular, our analysis showed that direct and indirect transmission routes shared only a handful of super-spreader farms, indicating that direct and indirect transmission risks were independent from each other. A major consequence of this observation is the need to account for both routes in the definition of contingency plans for the control of potential epidemics where indirect contacts represent an effective route of disease transmission. By considering only one of these transmission routes, we would miss a substantial part of the spreading pattern. This conclusion is strongly supported also by the analysis of infectious risk in the direct and indirect contacts networks. The *infection potential* was substantially lower for cattle movement (CM) compared to the veterinarians total (VT) network. This reflects the fact that farms may receive or move cattle only few times in a year and a large fraction of the farms in our database did not trade any animal during the study period. On the contrary, each veterinary practitioner visits a small set of farms on a regular basis, while veterinary officers have frequent visits to farms but visit the same farm only once a year on average. Farm *infection potentials* derived by using the network of indirect contacts is not correlated with that derived by using the network of direct contacts, thus reinforcing the finding that the transmission pathways of the two contact networks are remarkably different. The most striking result of our study, however, is that the *infection potential* derived by combining the networks of direct and indirect contacts is considerably larger than the one computed by using only cattle movements or the veterinary network (Fig 6). This suggests a synergistic effect between the networks of direct and indirect contacts: despite being sparse, direct contacts act as a bridge joining different clusters of potentially infectious contacts due to veterinarians. Despite movements of infected animals usually play a primary role in the spread of livestock epidemics (since they represent the most efficient transmission route between farms), our results highlighted the importance of considering indirect contacts to adequately model between-farm spread of infections. We showed that the combination of different pieces of information included in the *infection potential* metric is essential to understand the role of farms within the study system. In essence, the *infection potential* is the expression of the two fundamental components of the contact system, the contact structure and the temporal sequence of the potential infectious contacts [3,30,39]. In fact, as pointed out in many studies [4,37,38,41–43], traditional network metrics based on a static representation of the contacts between nodes can hide significant temporal patterns in epidemic network structures of fast spreading diseases, such as FMD. Conversely, the temporal sequence of contacts is crucial in defining the real risk of epidemic spread in a network. For this reason, we applied the concept of infection chains proposed by Dubé et al. [3] and extended by Konschake et al. [39], providing the estimate of the potential maximum size of epidemics in a network system. Here, as a measure of farm *infection potential*, we proposed the weighted product of in- times out-infection chains. This new metric aims to extend the concept of basic reproduction number for between-farm transmission provided by Woolhouse et al. [1], defined as the product of in-times out-degrees. Since the duration of the farm infectious period (γ) varies across different diseases, the stability of our results is indicative of the validity of the analysis with regard to a wide range of diseases. However, Konschake and colleagues [39] showed that in German swine livestock, risk ranking of farms according to *IC* was not stable with respect to variations in γ, in particular for γ shorter than 14 days. The different outcome of our study could be due to the structural differences between dairy and swine livestock industries.

We also analysed a weighted version of CM, VO and VP networks (where the links were weighted proportionally to the number of animals exchanged, in CM, and to the number of on-farm visits, in VO and VP). Similarly to the results in the unweighted networks, the measures of strength showed to be only partially correlated with the *infection potential*. However, in contrast with the unweighted cases (where VO and VP networks showed larger degree distributions than CM network), farms' strength distributions in the weighted networks showed similar patterns for direct contacts (CM) and for indirect contacts networks (VO and VP). This might be important for low-prevalence or poorly contagious diseases, in which the number of shipped animals or the recurrence of personnel-mediated contacts might strongly influence the probability of infection spread between farms.

Previous questionnaire-based works applied network analysis techniques on small networks of cattle exchanges and on-farm visits by veterinarians [10,16]. Similarly to their findings, our results showed that indirect contacts produced a more connected network compared to cattle movements. This finding was supported by the traditional static measures we applied to the investigated networks. The link density and the giant strongly connected component (GSCC) were more than one order of magnitude higher in VO and VP networks than in CM network, both in- and out-degree distributions showed longer tails, and the number of isolated farms was substantially greater in CM network, implying a higher number of farms reachable during an epidemics via indirect contacts than via direct contacts. For CM network, the observed value of GSCC was also lower than that observed elsewhere, such as in Scottish [29] and French herds [44]. This result is probably due to the spatial scale considered, since within-province animal exchanges only represent a fraction of the animals introduced in the dairy farms in Parma Province. On the other hand, contact frequency in CM network was slightly higher than in VO and VP networks, underlining a higher recurrence of these contacts. However, the VP network considered in our analysis might represent an underestimate of the real contact frequency, as the dataset used in the analysis only accounted for traceable visits in public datasets. In fact, the average number of monthly visits derived from our datasets was on the lower range than those observed in similar farm systems ([11,14,45]; see S1 Text for more details).

### On farm exposure to infection

Our analysis suggested that the network of veterinarian contacts is associated to the distribution of MAP positive farms in the Province of Parma, while the network of cattle movements is associated to the distribution of MAP positive farms when the farm system of the whole Emilia-Romagna region is considered. These outcomes can be interpreted as the effect of the different spatial scales on the observed processes. The provincial level represents a spatial scale that could properly fit the process of disease spread due to veterinary practitioners, because of their limited range of activity, and to veterinary officers who operate into sub-provincial public health districts. Conversely, the CM network at provincial level, which only looks at within-province animal exchanges, cannot take into account for the role in disease transmission of animals introduced from outside the province, which represent a significant fraction of the exchanges. Consequently, the relationship between infection status and animal exchanges becomes apparent only at a wider spatial scale, such as at the regional level, where more nodes (i.e. farms) and links (i.e. animal exchanged) are intercepted by the network.

We also showed that spatial clustering failed to explain the observed pattern of MAP infection (Table 2). Then, our analysis suggests that, at the investigated spatial scale, between-farm transmission was not significantly affected by spatial proximity among farms. The latter result is in agreement with the findings obtained by Ahlstrom et al. [46] on MAP distribution in Canada at intra-provincial scale by using single nucleotide polymorphisms identified through whole genome sequencing. From this finding, they suggested that human driven activities (such as cattle movements) are major drivers of MAP transmission at the herd level in contrast to spatially-localized transmission mechanisms (e.g. wildlife), which are crucial in the transmission of other pathogens, such as *Mycobacterium bovis* [46]. In a similar way, we suggest that, in addition to direct contacts, also indirect contacts can contribute to between-farm MAP spread at local level.

Similarly to our results, by analysing bulk tank milk samples, Garcia-Alvarez et al. [16] showed that the veterinary network could explain the difference in the infectious status of dairy farms for some strains of *Staphylococcus aureus*. However, unlike Garcia-Alvarez and colleagues [16], we did not know the temporal pattern of MAP infection across farms. Therefore, to account for MAP introductions occurred before the time period considered in the study, we also tested the significance of the relationship between network structures and infectious status by using contact data from years before 2013 and maintaining the infectious status of 2013. We found that the results were robust compared to the tested year, suggesting a high tendency of farms to maintain contacts with the same elements over time [41].

Our results suggested that between-farm transmission of MAP through fomites might be not negligible at local level. Consistent with our observation, Marcé et al. [32] mentioned the transfer of faeces, manure, slurry, and soiled forage as a viable route of pathogen introduction into farms. The transmission of MAP via contaminated environment has been already pointed out in several empirical works (specifically, via footwear [24], via shared equipment [25], and via settled-dust [48]) and theoretical models of within-farm transmission [49,50].

In this work, we defined the positive/negative infection status of farms for *paratuberculosis* using data obtained by Ricchi et al. [21] through real-time PCR on bulk tank milk samples. Real-time PCR techniques on bulk tank milk display only intermediate sensitivity in detecting MAP in cattle herds [47], in particular because they do not detect not-excreting infected animals. It follows that the obtained prevalence represents an underestimate of the between-farm true prevalence in the area. However, for the purpose of our analysis, the major focus was the contamination status of farms; in that sense, the real-time PCR assessment of the presence of MAP in the herd environment is functional to estimate the ability of the farms to spread the infection, especially in the case of fomite contaminations and in the short run.

## Conclusions

Our results highlight the urgency of further defining the indirect contacts between farms established by operators like veterinarians, milk, feed, and slaughterhouse trucks, and artificial insemination technicians, in addition to estimates of non-recordable movements such as neighbours and service providers, that even if less infectious, would be more frequent farm visitors [51]. The availability of personnel visits data would improve the surveillance and control of epidemics through modelling approaches. In particular, this could make it possible to effectively tackle diseases among which we recognize some of the most devastating threats to the modern livestock industry, above all, foot-and-mouth disease and avian influenza.

## Supporting Information

S1 Text Supplementary analyses. Including: data descriptive statistics and analysis, sensitivity analysis on the contamination period, weighted network analysis, network structure and farm exposure to infection using different assumptions, infection chains and infection potential.
(PDF)

S1 Data Dataset used in the study. This includes three CSV files reporting a configuration of the contacts list for the considered networks (cattle movement, veterinary officers, and veterinary practitioners).
(ZIP)
