# Supplementary material for: The Potential Role of Direct and Indirect Contacts on Infection Spread in Dairy Farm Networks
Source: PLoS Comput Biol. 2017 Jan 26;13(1):e1005301. doi: 10.1371/journal.pcbi.1005301 (PMC5268397; doi:10.1371/journal.pcbi.1005301)
Supplement: S1 Text — Including: data descriptive statistics and analysis, sensitivity analysis on the contamination period, weighted network analysis, network structure and farm exposure to infection using different assumptions, infection chains and infection potential. (PDF) [file pcbi.1005301.s001.pdf]

S1 Text: supplementary analyses

The potential role of direct and indirect contacts on epidemics in dairy farms networks

G. Rossi, G. A. De Leo, S. Pongolini, S. Natalini, L. Zarenghi, M. Ricchi, L. Bolzoni

S1.1. Data descriptive statistics and analysis

S1.1.1. Within-day visits order

As explained in the main text (*Veterinarians movement data* in *Materials and Methods* section) our dataset lacked of the exact time at which visits were performed by veterinarians, and consequently the order with which both veterinary officers (VO) and veterinary practitioners (VP) visited the farms was unknown. The distribution of the number of farm visited within the same day by the same veterinarian was showed in Figure S1.1.1. However, the order was needed in order to draw the links to build the network, and to calculate the *infection chains*. To do so, we computed the daily itineraries by assuming a minimum-length itinerary travelled by the veterinarians through package “TSP” in R (Hahsler and Hornik, 2007). As neither veterinarians’ starting positions were available, there were at least two possible minimum-length paths for each itinerary, one the exact reverse of the other. This lead to a large number of possible network configurations. In fact, if we define *D* (= 3881) as the number of observed itineraries with more than one farm, the total number of configuration is given by 2^D. However, veterinarians generally visit a limited number of farms and itineraries tend to be repeated similarly over time. This make it possible to catch most of the variability with by a low number of simulations. Preliminary results showed that 50 randomly extracted configurations are able to catch the variability of the potential network configurations. Moreover, this variability tend to be substantially reduced if we assume values of contamination period (*h*, see main text, *Veterinarians movement data* in *Materials and Methods* section) higher than 0 (*h* sensitivity analysis results is reported in S1.2

section). One extracted configuration of the 3 networks (cattle movements, CM, VO, and VP) is showed in Figure S1.1.2.

S1.1.2. Cattle movements

The number of cattle exchanged within the dairy farm system of the Province of Parma during year 2013 was 16,647, corresponding to a daily average [median] of 45.61 [39]. The time series of the number of moved individuals on a daily and weekly basis are shown in Figure S1.1.3.

S1. 1.3. Veterinary officers

The average [median] per capita farm visits for the veterinary officers (VO) in 2013 was 130.5 [143], ranging from 1 to 267 visits per officer. The overall mean [median] number of daily visits within the farm system was 17.87 [16] (range 0–110). Each dairy farm received a mean [median] of 4.83 [4] visits per year (range 0–54). The daily number visits during year 2013 was showed in Figure S1.1.4(a). A seasonal pattern was clearly identifiable, with peaks in the number of daily visits in spring (March-April) and autumn (October). In Figure S1.1.5 we showed the number of farms subdivided in classes as a function of the number of monthly visits received by VOs. The average [median] number of monthly visits per farm was 0.40 [0.33], and they ranged from 0 to 4.5.

The in- and out- degree were significantly correlated between each other, as among the 20 simulations of the network configurations (see S1.1 for details) the median Kendall's τ observed was 0.50 (range 0.47–0.55, $p < 0.01$ in all cases).

S1.1.4. Veterinary practitioners

Veterinarian practitioners (VP) visits data derived from three different datasets: the list of prescribed drugs, animal tissue droppings at the Istituto Zooprofilattico Sperimentale, and the inspections

subcontracted to VP (see main text for further details). However, since on-farm visits by VP are not always followed by one of the previous reporting, the available total dataset is an under-representation of the total number of VP visits.

The total number of VPs recorded was 203, and the number of recorded visits was 14,053. The mean [median] number of recorded visits per VP was 69.2 [24], ranging from 1 to 545. The mean [median] number of daily visits within the overall system was 38.5 [43] (range 1–78), as it was represented in Figure S1.1.4(b). In contrast with VO visits, there was no clear seasonal pattern in the number of daily visits.

We showed in Figure S1.1.5 the number of farms subdivided in classes as a function of the number of monthly visits by VPs. The average [median] VP monthly visits per farms was 0.87 [0.5], and it ranged from 0 to 10.67. Furthermore, 292 farms had zero VP visits recorded during 2013. Data from the literature (Table S1.1.1) showed estimates within the same range, but with generally higher averages (Bates et al., 2001; Mc Reynolds et al., 2014; Matteucci and Massirio, 1999; Noremark et al., 2013; Richert et al, 2013).

The VP in- and out- degree were highly correlated between each other, as among the 50 simulations the median Kendall's τ observed was 0.70 (range 0.38–0.57, $p < 0.01$).

S1.2. Sensitivity analysis on the contamination period, *h*

The contamination period, h, represents the time span in which an operator remain contaminated by a pathogen after a visit into an infected farm. This period length depends on several factors, including the ability of the pathogen to survive on fomites and in the environment. In our analysis we assumed that only consecutive visits by the same operator occurring during the same day could potentially lead to an infection event, corresponding to $h = 0$. Thus, potentially infective links were assumed to exist from a farm visited in a given day to a farm visited later the same day by the same operator (see the

main text, *Veterinarians movement data* in *Materials and Methods* section) for further details on the assumptions on within-day visits order). However, for different diseases (such as Foot-and-Mouth Disease, bovine tuberculosis, and paratuberculosis) the survivor of the pathogen in fomites and environment can be substantially longer, in particular in winter conditions (Sanson et al., 1993; Morris et al., 1994; Whittington, et al. 2004). Therefore, in order to assess the effect of the h value on the characteristics of the VO and VP networks, we performed a sensitivity analysis on structural network properties and on farms degree distributions for different values of h; specifically, $h = 0$ (benchmark), 7, and 14 days.

In the VO network, all structure measures dramatically increased for higher values of h (see Table S1.2.1), suggesting an even stronger role for indirect transmission respect to the benchmark scenario. In particular, from 0 to 7 days, and from 7 to 14 days, the median of the links density increased of ≈ 300% and 39%, respectively. In the same settings, the GSCC increased of 36% and 1% respectively, while the average contacts frequency increased of 9% and 8%, respectively. To quantify the variations in the farms degree, we considered the median (and percentiles) over the 20 simulations of the farms average degree. The obtained values were reported in Table S1.2.2.

The median [5th–95th percentile] average farms degree in the VO network (benchmark case, $h = 0$) was 6.55 [6.36–6.76], and the maximum observed values of in- (k_I) and out-degree (k_O) were respectively 45 and 44. By setting h to 7 days the median [5th–95th percentile] average degree increased to 27.86 [27.65–28.04] and the maximum observed k_I and k_O to 106 and 101. By setting h to 14 days the median [5th-95th percentile] average degree increased to 38.72 [38.57–38.80] and the maximum observed in- and out-degree to 142 and 124. The differences among the farms degree distributions $P(k_I)$ and $P(k_O)$ in VO network for different values of h (0, 7, and 14 days) are showed in Figure S1.2.1 (panels *a* and *b*, respectively).

A similar pattern was observed for the VP networks. In particular, for an increase of h from 0 to 7

days, and from 7 to 14 days the links density increased of ≈ 500% and 43%, respectively. In the same conditions, the GSCC increased of 38% and 1%, while the average contacts frequency increased of 66% and 18%, respectively (see Table S1.2.1).

The VP network at *h* equal to 0 showed a lower degree with respect to the VO one. In fact, the median [5th-95th percentile] average farms degree over the 20 simulations was 3.90 [3.86-3.94], and the maximum observed values of in- (k_I) and out-degree (k_O) were respectively 35 and 34. As in the VO networks, the degree distributions of the VP network ($P(k_I)$ and $P(k_O)$) are showed in Figure S1.2.1 (panels *c* and *d*, respectively). By setting *h* to 7 days the median [5th-95th percentile] average degree observed was 24.00 [23.96-24.06] and the maximum observed k_I and k_O respectively 158 and 131. By setting *h* to 14 days the median [5th-95th percentile] average degree was 34.33 [34.26-34.38] and the maximum in- and out-degree respectively 204 and 193. While in both cases the average degree was lower in the VP network with respect to VO ones, both maxima observed degrees were higher in VP networks (see Table S1.2.2).

In conclusion, we observed how the choice a given contamination period *h* could strongly influence the network basic structure as showed by the analysis of measures such as degree, link density, and GSCC.

S1.3. Weighted network analysis

As we described in the main text (see *Materials and Methods*) we built an unweighted and a weighted version of the three networks (i.e.: cattle movement, CM, veterinary officers, VO, and veterinary practitioners, VP). In VO and VP weighted networks, the links' weight corresponded to the yearly aggregated frequency of contacts, while in CM network it corresponded to the number of exchanged animals. In this section of the S1 Text we showed the results of the analyses on the weighted contact networks. Following the nomenclature contained in the work of Barrat and colleagues (2004), we will refer to farms strength (S) as the sum of all incoming (in-strength, S_I) and (out-strength, S_O) outgoing

links' weights.

In Figure S1.3.1 we showed the distribution of the in- and out-strength for the CM, VO, and VP weighted networks. For VO and VP, we set h to 0, i.e. assuming that only visits within the same day could potentially lead to disease transmission.

In CM network farms S_I and S_O were partially correlated among each other (Kendall's $\tau = 0.37$, $p < 0.01$). A similar outcome was observed for the VO and VP networks, in which farms S_I and S_O were significantly correlated (VO: median [range] Kendall's $\tau = 0.49$ [0.48–0.54], $p < 0.01$; VP: median [range] Kendall's $\tau = 0.70$ [0.69–0.71], $p < 0.01$). The similar in- and out-strength due to VP visits was probably due to the visits recurrence of each practitioner in a given pool of farms, while VO visits did not experienced a similar recurrence effect.

As showed in Table S1.2.2, CM weighted network had both higher average strength and higher maximum values, with respect to VO e VP networks. Among the two veterinarian networks, VO and VP, only in the second one the strength had substantially higher values than the degree in the unweighted network. This was due to the recurrence of each single practitioner visits on a limited number of farms, whereas each officers, on average, visited an higher number of farms but fewer times, leading to a low number of links recurrence. This effect is observed despite the under-reported number of VP visits in our datasets (in contrast to the VO dataset where all visits were reported). This suggest that strength differences between VP network with respect to VO could be even stronger. However, degree and strength were generally highly correlated. For the CM network the Kendall's τ calculated among farms S_I and k_I was 0.79 ($p < 0.01$), while among S_O and k_O was 0.81 ($p < 0.01$). The Kendall correlations between S and k were higher for the VO network, 0.94 and 0.94 respectively ($p < 0.01$), as well as for the VP network, 0.97 and 0.97 respectively (p's < 0.01).

In Figure S1.3.2 we showed the in- and out-strength distributions, $P(S_I)$ and $P(S_O)$, for different values of contamination period, h. By assuming larger h values, both VO and VP networks average degree

became higher than CM network, although the maximum strength recorded in VO network remain lower than CM network.

S1.4. Network structure and farm exposure to infection using different assumptions

In this section, we tested for the cattle exchange networks in the province of Parma and in Emilia-Romagna region, where data were available for multiple years, whether contact networks built by using data from years 2010, 2011, and 2012 can explain the differences in the infectious state by *Mycobacterium avium* subsp. *paratuberculosis* (MAP) detected in 2013. We performed these supplementary analyses to test whether the conclusions presented in the main text (based on the 2013 contact networks) held by assuming the MAP introduction in the farms having occurred before the time period considered in the study (see *Farm exposure to infection* in *Results* section). Analogously to the main text analyses, Figures S1.4.1 (Parma province) and S4.2 (Emilia-Romagna region) show that the mean exposures of MAP positive farms (blue dots, E_I) in the cattle movement networks were higher than those of MAP negative farms (red dots, E_S) for the years considered in the analysis. In addition, we found that the mean exposures of MAP positive farms in the Parma province networks were not significantly higher than in randomly generated networks (blue vertical bars, Figure S4.1), while the mean exposure of MAP positive farms in the Emilia-Romagna region networks were significantly higher than in randomly generated networks (blue vertical bars, Figure S1.4.2).

In addition, we performed a sensitivity analysis of the exposure results with respect to the contamination period (h) in the veterinary networks. Analogously to the main text analysis, Figure S1.4.3 shows the mean exposure of MAP positive farms (blue dots, E_I) in the veterinary network was higher than in MAP negative farms (red dots, E_S) also for h = 7 days. In addition, we found that the mean exposure of MAP positive farms in the veterinary network was significantly higher than in randomly generated networks, $p = 0.033$ (blue vertical bars, Figure S1.4.3).

S1.5. Infection chains and infection potential

As pointed out in the main text (see *Define infection potential and super-spreaders* in *Materials and Methods* section), in order to compute the infection chains (*IIC* and *OIC*) and, thus, the infection potential (ρ), we assumed a farm infectious period (γ) of 14 days. This assumption followed two main considerations: (*i*) according to Konschake and colleagues (2013), $\gamma = 14$ was the threshold value above which the *IC* measure was stable; and (*ii*) the assumed infectious period was congruent with that observed for diseases such as the Foot-and-Mouth Disease (Bates et al., 2003). In this section, we performed a sensitivity analysis on the effects of different values of γ (specifically: 3, 7, 14, 21, and 28 days) on *ICs* estimates.

In Figure S1.5.1 we showed four colour-plots representing the correlation among the *IICs* values obtained with different values of γ for each farms in the CM, VO, VP and veterinarians total visits (VT) networks. All correlations among the five cases were significant, in particular for CM network (all combination τ coefficients higher than 0.93) and for VP network (minimum τ value 0.79), while *IIC* correlations for VO and VT networks were less strong (minimum τ values 0.71 and 0.60, respectively). The results for *OIC* (Figure S1.5.2) gave similar results, with minimum correlation coefficient values observed of 0.93, 0.79, 0.73 and 0.59, respectively for CM, VP, VO, and VT networks. However, as showed in Figure S1.5.3, infection potential ρ showed a higher sensitivity on γ, determining lower correlations (minimum correlation coefficient values observed of 0.78, 0.74, 0.64 and 0.48, respectively for CM, VP, VO, and VT networks). These results showed that farms rankings, according to infection chains and infection potential, were robust to relatively small changes in infectious period, a parameter that largely is disease specific. However, as the gap among infectious periods spread, the correlation was substantially reduced, suggesting that a unique measure for every diseases could lead to misleading rankings. This consideration is important in particular when targeted

surveillance and control measures are based upon those farms rankings.

Finally, we computed the correlation between the infection potential -ρ- and the degree, showed in Figure S1.5.4, and between ρ and strength, showed in Figure S1.5.5. In all cases, Kendall correlation coefficients ranged from $\tau = 0.6$ to $\tau = 0.8$, and they generally showed stability or a decreasing pattern for increasing values of γ. Correlations in the CM network between degrees and strengths and ρ increased for increasing values of γ, but were also the lowest with respect to the other networks. These analyses suggest that, although degree or strength are good centrality measure in farms network, they do not completely capture the effect of the temporal sequence of contacts. On the other hand, this effect is included in the infection potential measure. Moreover, these results showed how the information loss was stronger in the case of CM network. Considering that cattle movements are the most effective transmission route for disease within farms network, that loss of information can be crucial for the outcome of many analysis.

Software

All statistical analyses, network analyses and drawings were performed using the software R with "igraph", "tnet", "reshape2", and "plyr" packages (Csardi and Nepusz, 2006; Opsahl and Panzarasa, 2009; R Core Team, 2015; Wickham, 2011, 2007). Spatial clustering analysis, including the q-nearest neighbour test, was performed using packages "spatstat" (Baddeley and Turner, 2005) and "smacpod" (French, 2014).

References:

Baddeley, A., Turner, R., Mateu, J., Bevan, A., 2013. Hybrids of Gibbs Point Process Models and Their Implementation. J. Stat. Softw. 55, 1–43.

Barrat, A., Barthélemy, M., Pastor-Satorras, R., Vespignani, A., 2004. The architecture of complex weighted networks. Proc. Natl. Acad. Sci. U. S. A. 101, 3747–3752.

Bartley, L., Donnelly, C.A., Anderson R. M., 2002. Review of foot-and-mouth disease virus survival in animal excretions and on fomites. Vet. Rec. 667–670.

Bates, T.W., Thurmond, M.C., Carpenter, T.E., 2001. Direct and indirect contact rates among beef, dairy, goat, sheep, and swine herds in three California counties, with reference to control of potential foot-and-mouth disease transmission. Am. J. Vet. Res. 62, 1121–1129.

Csardi, G., Nepusz, T., 2006. The igraph software package for complex network research. InterJournal, Complex Systems 1695.

French, J., 2014. smacpod: Statistical Methods for the Analysis of Case-Control Point Data. https://cran.r-project.org/web/packages/smacpod/ .

Hahsler, M., Hornik, K., 2007. TSP -- Infrastructure for the traveling salesperson problem. J. Stat. Softw. 23, 1–21.

Kao, R.R., Danon, L., Green, D.M., Kiss, I.Z., 2006. Demographic structure and pathogen dynamics on the network of livestock movements in Great Britain. Proc. Biol. Sci. B 273, 1999–2007.

Konschake, M., Lentz, H.H.K., Conraths, F.J., Hövel, P., Selhorst, T., 2013. On the robustness of in- and out-components in a temporal network. PLoS One 8, e55223.

Matteucci, L., Massirio, I., 1999. Afta Epizootica: diffusione e fattori di rischio in allevamento. L'Osservatorio 2, 4–15.

McReynolds, S.W., Sanderson, M.W., Reeves, A., Sinclair, M., Hill, A.E., Salman, M.D., 2014. Direct and indirect contact rates among livestock operations in Colorado and Kansas. J. Am. Vet. Med. Assoc. 244, 1066–1074.

Morris, R.S., Pfeiffer, D.U., Jackson, R., 1994. The epidemiology of Mycobacterium bovis infections. Vet. Microbiol. 40, 153–177.

Nöremark, M., Frössling, J., Lewerin, S.S., 2013. A survey of visitors on Swedish livestock farms with reference to the spread of animal diseases. BMC Vet. Res. 9, 184.

Opsahl, T., Panzarasa, P., 2009. Clustering in weighted networks. Soc. Networks 31, 155–163.

R Core Team, 2015. R: A Language and Environment for Statistical Computing. The R Foundation for Statistical Computing. Vienna, Austria.

Richert, R.M., Cicconi, K.M., Gamroth, M.J., Schukken, Y.H., Stiglbauer, K.E., Ruegg, P.L., 2013. Management factors associated with veterinary usage by organic and conventional dairy farms. J. Am. Vet. Med. Assoc. 242, 1732–1743.

Sanson, R.L., Struthers, G., King, P., Weston, J.F., Morris, R.S., 1993. The potential extent of transmission of foot-and-mouth disease: a study of the movement of animals and materials in Southland, New Zealand. N. Z. Vet. J. 41, 21–28.

Wasserman, S., Faust, K., 1994. Social network analysis: Methods and applications. Cambridge university press. Cambridge, UK.

Whittington, R.J., Marshall, D.J., Nicholls, P.J., Marsh, A.B., Reddacliff, L.A., 2004. Survival and dormancy of *Mycobacterium avium* subsp. *paratuberculosis* in the environment. Appl. Environ. Microbiol. 70, 2989–3004.

Wickham, H., 2007. Reshaping Data with the {reshape} Package. J. Stat. Softw. 21, 1–20.

Wickham, H., 2011. The Split-Apply-Combine Strategy for Data Analysis. J. Stat. Softw. 40, 1–29.

Tables:

Reference	#visits	#farms	visits/farm	Time frame (days)	visits/ month/farm
Mettucci and Massirio, 1999	-	-	1.35	14	2.89
Noremark et al., 2013	281	482	0.58	14	1.25
Bates et al., 2001	-	-	2.35	30	2.35
Mac Raynolds et al., 2014	-	-	44.17	365	3.63
Richert et al., 2013	682	149	4.58	120	1.14
Current study	*20577*	*1349*	*15.25*	*365*	*1.25*

Table S1.1.1: The number of veterinarian visits per farm in five studies published in the scientific literature, including the current study. All selected studies referred to visits on dairy farms.

**Network**		**CM**		**VO**			**VP**	
h		-	0	7	14	0	7	14
Links density	Median	0.0008	0.0049	0.0207	0.0287	0.0029	0.0178	0.0255
	5th percentile	-	*0.0047*	*0.0205*	*0.0286*	*0.0029*	*0.0178*	*0.0254*
	95th percentile	-	*0.0050*	*0.0208*	*0.0288*	*0.0029*	*0.0179*	*0.0255*
GSCC (% on the network)	Median	1.33	67.75	92.22	93.25	54.26	75.17	76.13
	5th percentile	-	*35.62*	*91.99*	*93.11*	*53.44*	*75.09*	*76.06*
	95th percentile	-	*70.33*	*92.37*	*93.33*	*54.87*	*75.24*	*76.13*
Contacts frequency	Median	2.1	1.19	1.30	1.40	1.19	1.97	2.33
	5th percentile	-	*1.15*	*1.29*	*1.40*	*1.18*	*1.97*	*2.32*
	95th percentile	-	*1.22*	*1.31*	*1.41*	*1.20*	*1.98*	*2.33*

Table S1.2.1: Network measures (links density, GSCC, and contacts frequency) for cattle movement (CM) network, veterinary officers (VO) network (median, 5th and 95th percentile over 20 simulations), and veterinary practitioners (VP) network (median, 5th and 95th percentile over 20 simulations). The measures for VO and VP networks were calculated assuming three values of *h* (0, 7 and 14 days).

Network		CM	VO			VP		
h		-	0	7	14	0	7	14
K_I	Mean	1.06	6.55 (.36-.76)	27.86 (.65-28.04)	38.71 (.57-.80)	3.90 (.86-.94)	24.00 (23.96-.06)	34.33 (.26-.38)
	SD	*1.67*	*6.99*	*20.88*	*26.92*	*5.07*	*29.25*	*42.85*
	Max	*15*	*45*	*106*	*142*	*35*	*158*	*204*
K_O	Mean	1.06	6.55 (.36-.76)	27.86 (.65-28.04)	38.71 (.57-.80)	3.90 (.86-.94)	24.00 (23.96-.06)	34.33 (.26-.38)
	SD	*1.86*	*6.96*	*20.77*	*26.50*	*5.05*	*29.06*	*42.36*
	Max	*15*	*44*	*101*	*124*	*34*	*131*	*193*
S_I	Mean	12.34	7.78 (.78-.78)	36.18 (.13-.20)	54.31 (.38-.35)	6.46 (.46-.46)	47.34 (.30-.38)	79.82 (.80-.86)
	SD	*38.70*	*9.08*	*30.62*	*43.72*	*6.27*	*63.43*	*106.60*
	Max	*413*	*79*	*175*	*306*	*60*	*620*	*1017*
S_O	Mean	12.34	7.78 (.78-.78)	36.18 (.13-.20)	54.31 (.38-.35)	6.46 (.46-.46)	47.34 (.30-.38)	79.82 (.80-.86)
	SD	*37.40*	*9.12*	*30.90*	*43.27*	*6.47*	*59.48*	*95.40*
	Max	*469*	*85*	*180*	*253*	*60*	*448*	*540*

Table S1.2.2: In-degree, out-degree, in-strength, and out-strength values in three networks: cattle movement (CM), veterinary officers (VO), and veterinary practitioners (VP). For VO and VP networks we included the three *h* value cases (*h* = 0, 7 and 14 days). For each measure we reported the network mean value (the median, 5th percentile, and 95th percentile among the 20 simulations), the standard deviation (SD) and the maximum observed value.

Figures:

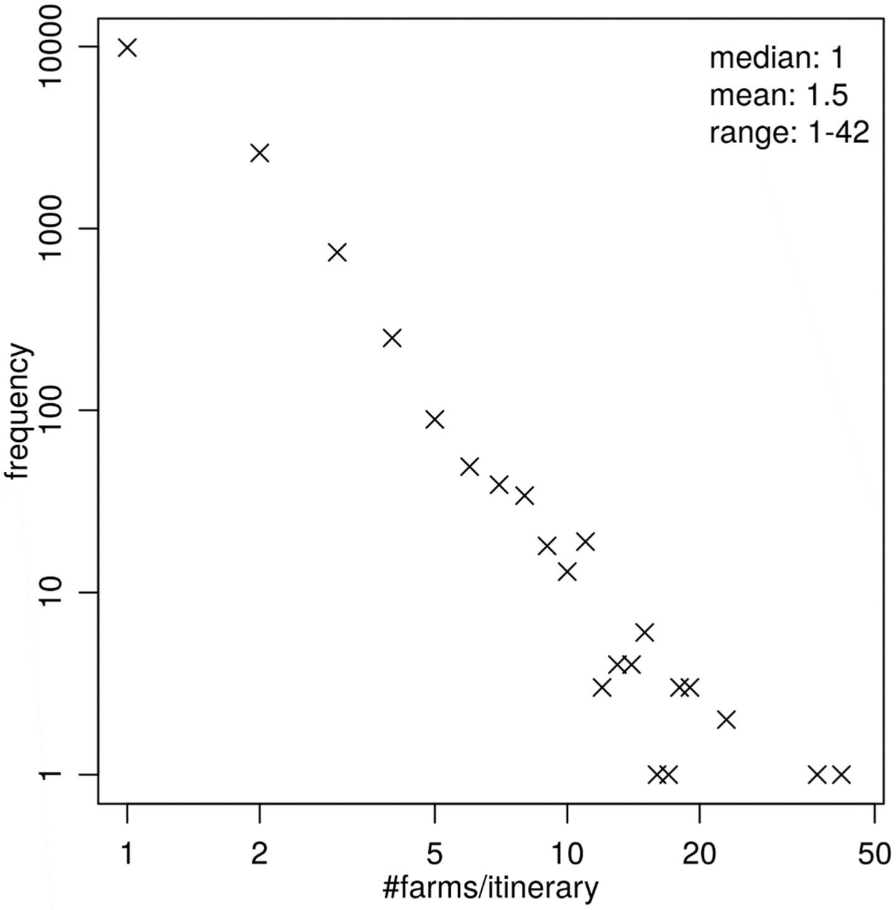

Figure S1.1.1: Distribution (log-log scale) of the number of farms in each daily itinerary (i.e. visited by a veterinarian during the same day).

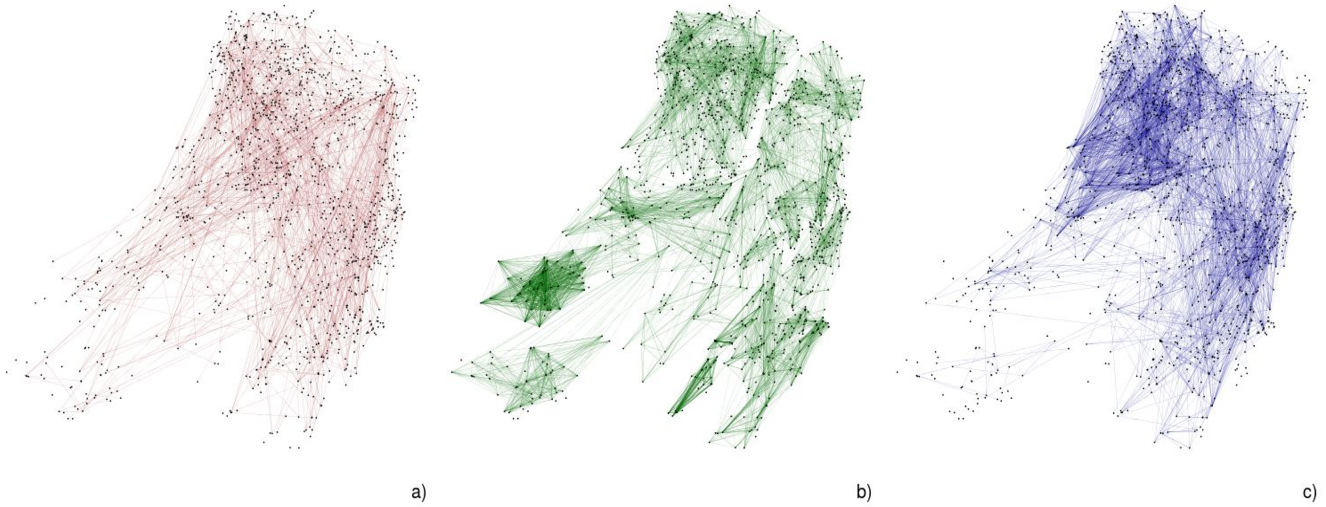

Figure S1.1.2: Province of Parma Dairy farms network. a) Cattle movements (CM) network (red), b) veterinary officers (VO) network (green, 1 configuration), and c) veterinary practitioners (VP) network (blue, 1 configuration).

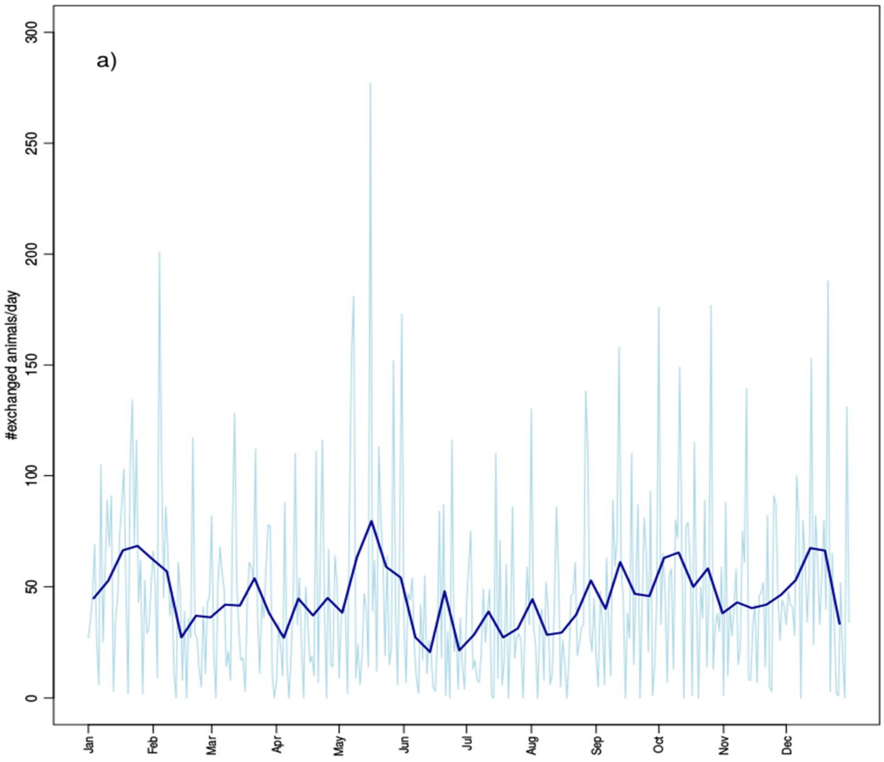

Figure S1.1.3: Year 2013 time series for number of daily (light blue) and mean weekly (dark blue) moved animals.

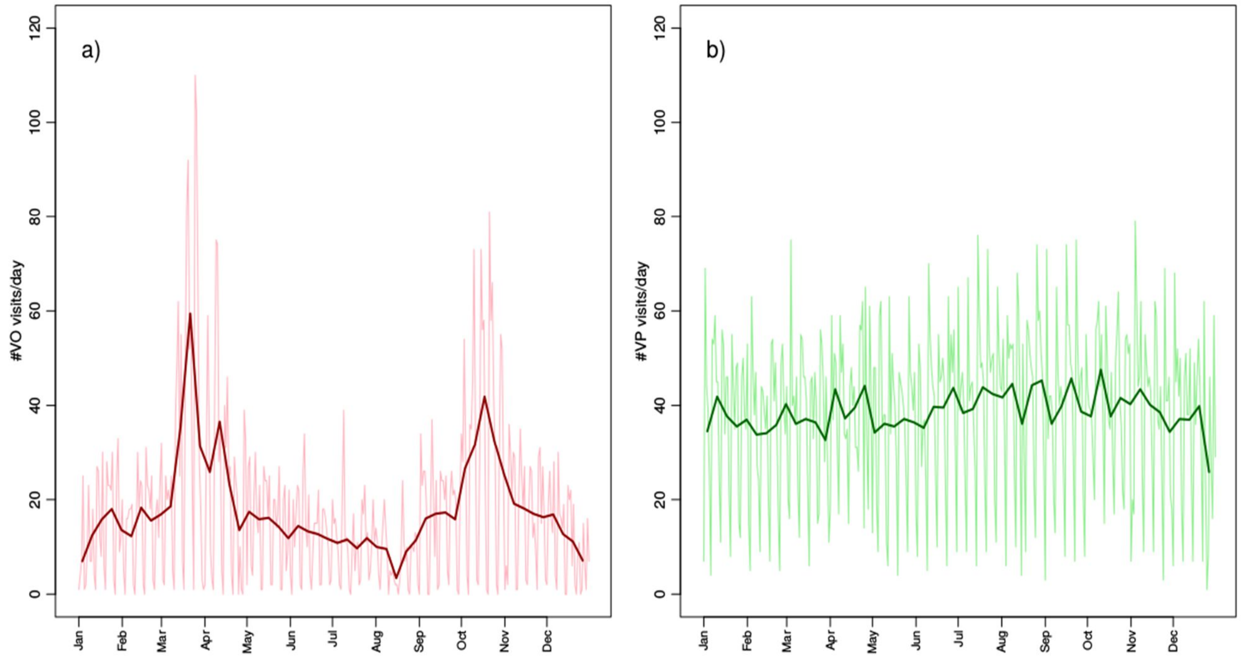

Figure S1.1.4: Year 2013 time series for: a) number of daily (light red) and mean weekly (dark red) VO visits, and b) number of daily (light green) and mean weekly (dark green) VP visits.

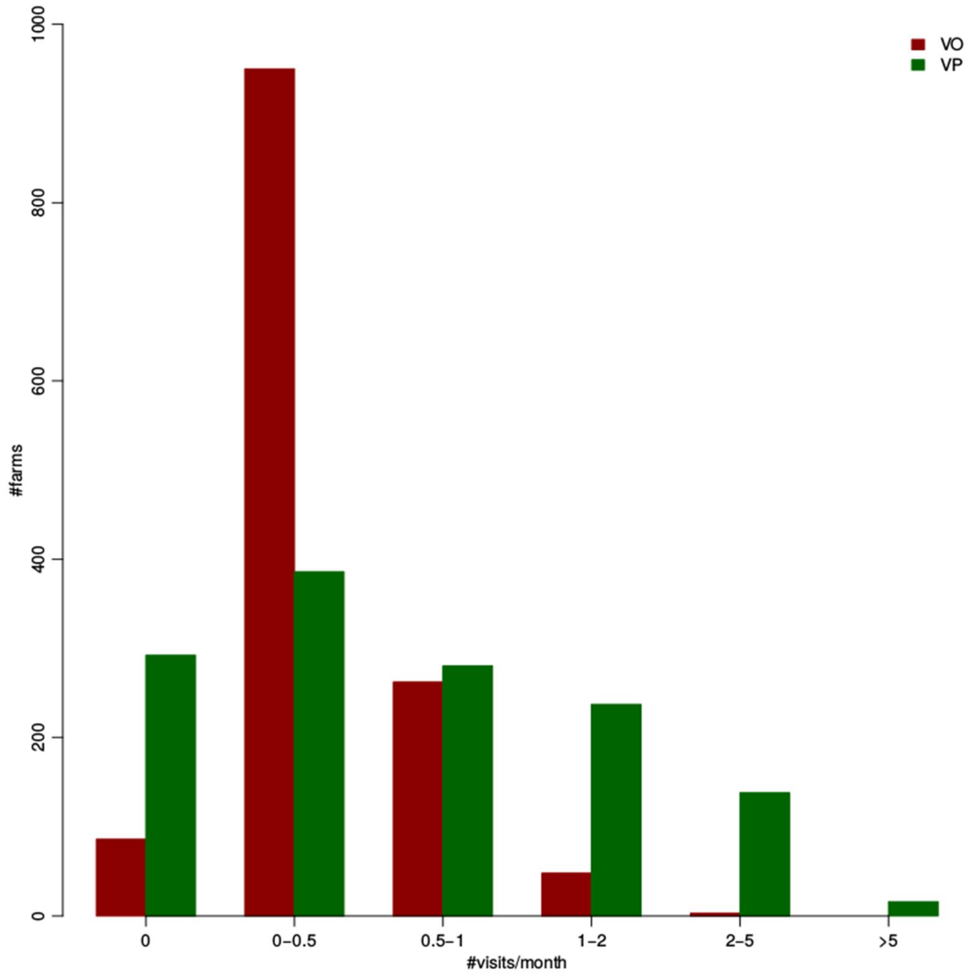

Figure S1.1.5: Number of dairy farms within the Province of Parma divided into classes as a function of the number of monthly visits received by veterinary officers (VO, red bars) and veterinary practitioners (VP, green bars).

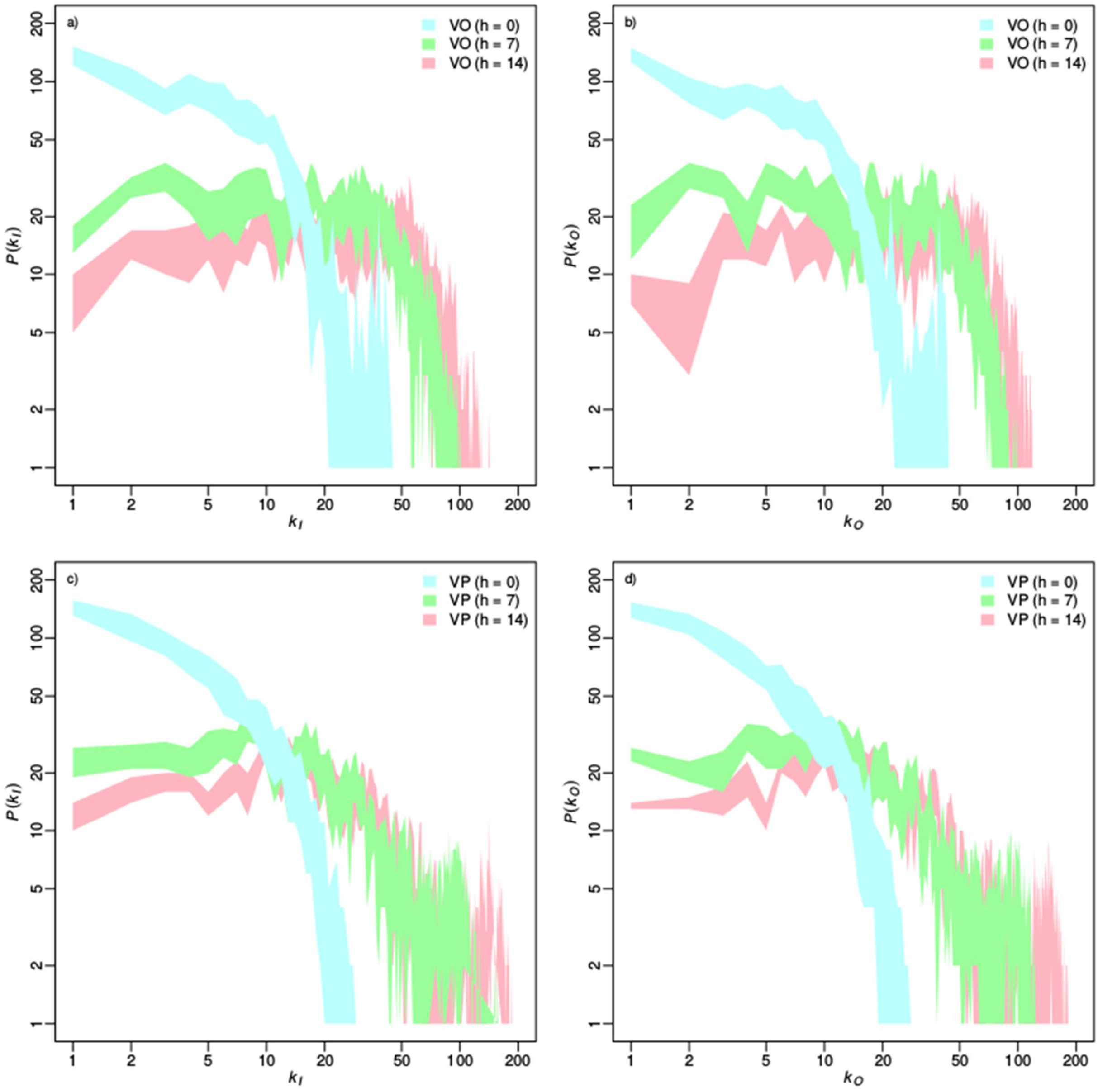

Figure S1.2.1: Farms in- and out-degree distributions (log-log scale) of veterinary officers (VO) and veterinary practitioners (VP) unweighted networks, at different values of h: 0 (blue), 7 (green) and 14 (red) days. The four panels represented: a) k_I in VO network, b) k_O in VO network, c) k_I in VP network, and d) k_O in VP network. The areas represented the values among 20 simulations.

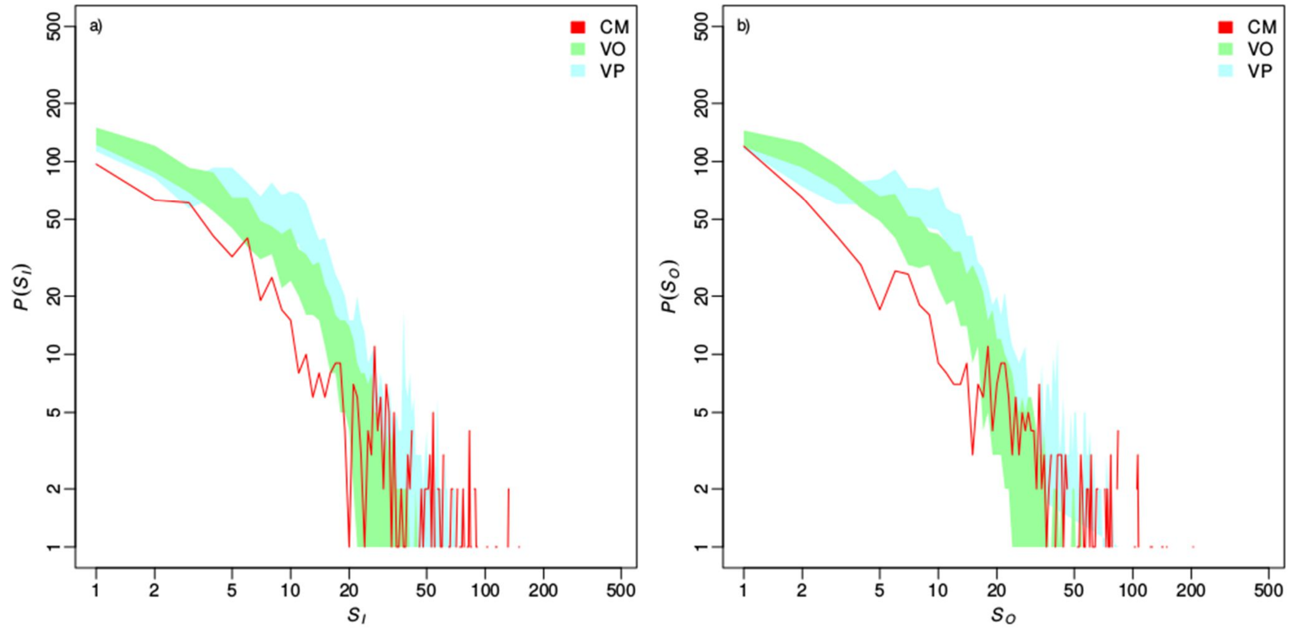

Figure S1.3.1: Farms in- (panel a) and out- (panel b) strength distributions (log-log scale) of cattle movement (CM, red), veterinary officers (VO, green) and veterinary practitioners (VP, blue) weighted networks. VO and VP networks were built considering $h = 0$. The areas in VO and VP cases represented the values among 20 simulations.

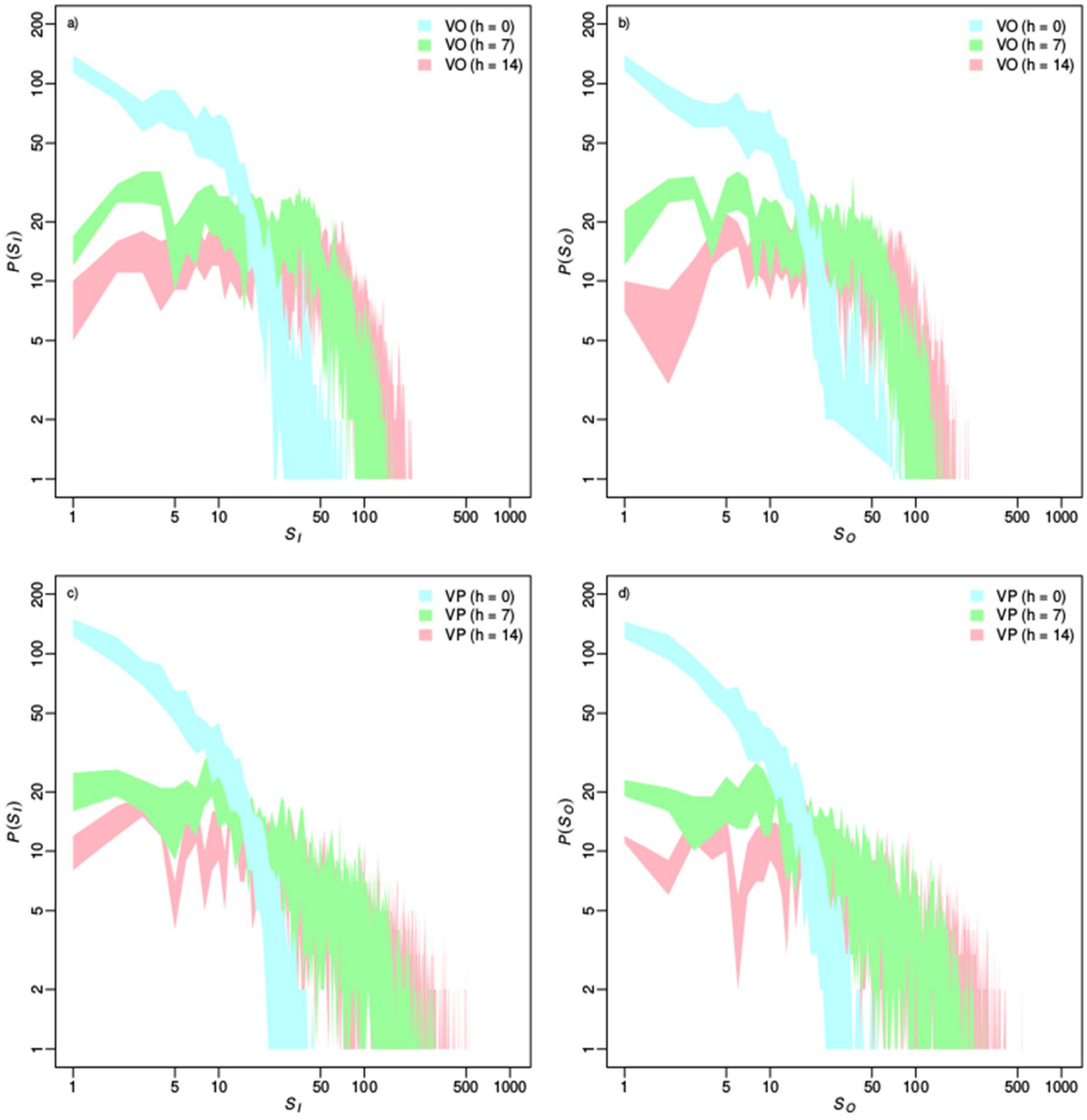

Figure S1.3.2: Farms in- and out-strength distributions (log-log scale) of veterinary officers (VO) and veterinary practitioners (VP) weighted networks, at different values of h: 0 (blue), 7 (green) and 14 (red) days. The four panels represented: a) S_I in VO network, b) S_O in VO network, c) S_I in VP network, and d) S_O in VP network. The areas represented the values among 20 simulations.

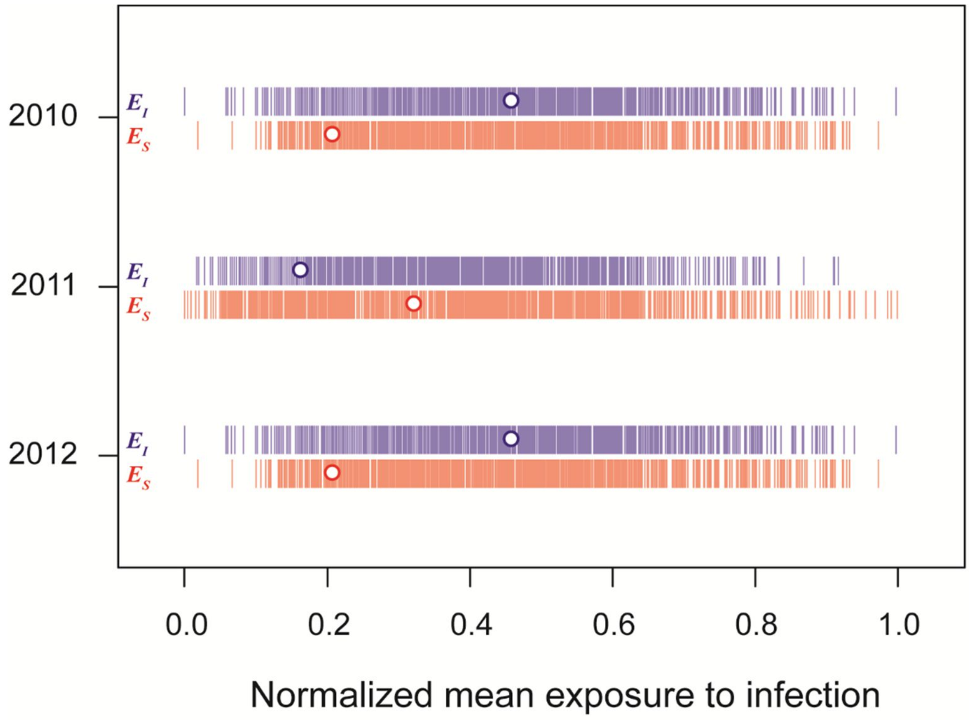

Figure S1.4.1: Mean exposure to MAP-positive (blue dots, E_I) and MAP-negative (red dots, E_S) farms normalized between zero and one (respect to their infectious status in 2013), in cattle movement networks of the Parma Province for years 2010, 2011, and 2012. Vertical bars represent the mean exposure to MAP in random networks with the same strength distributions of the observed networks.

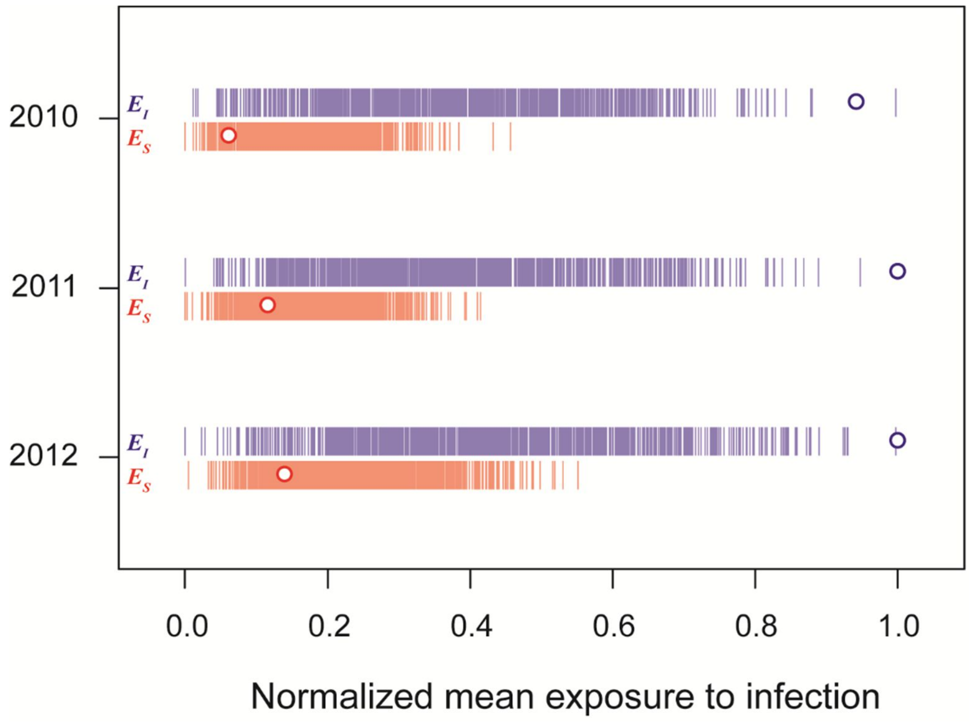

Figure S1.4.2: Mean exposure to MAP-positive (blue dots, E_I) and MAP-negative (red dots, E_S) farms normalized between zero and one (respect to their infectious status in 2013), in cattle movement networks of the Emilia-Romagna region for years 2010, 2011, and 2012. Vertical bars represent the mean exposure to MAP in random networks with the same strength distributions of the observed networks.

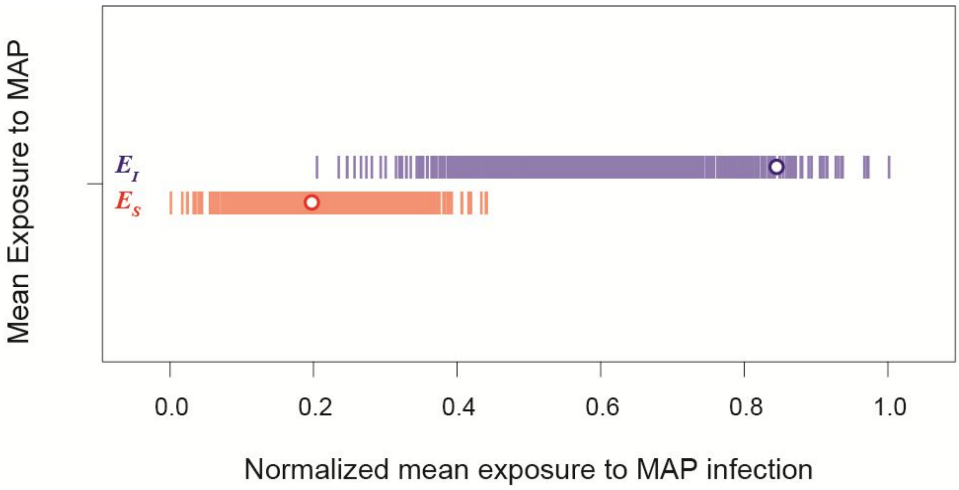

Figure S1.4.3: Mean exposure to MAP-positive (blue dots, E_I) and MAP-negative (red dots, E_S) farms normalized between zero and one in the veterinary network of Parma province under the assumption of a contamination period h = 7 days. Vertical bars represents the mean exposure to MAP in random networks with the same strength distributions of the observed networks.

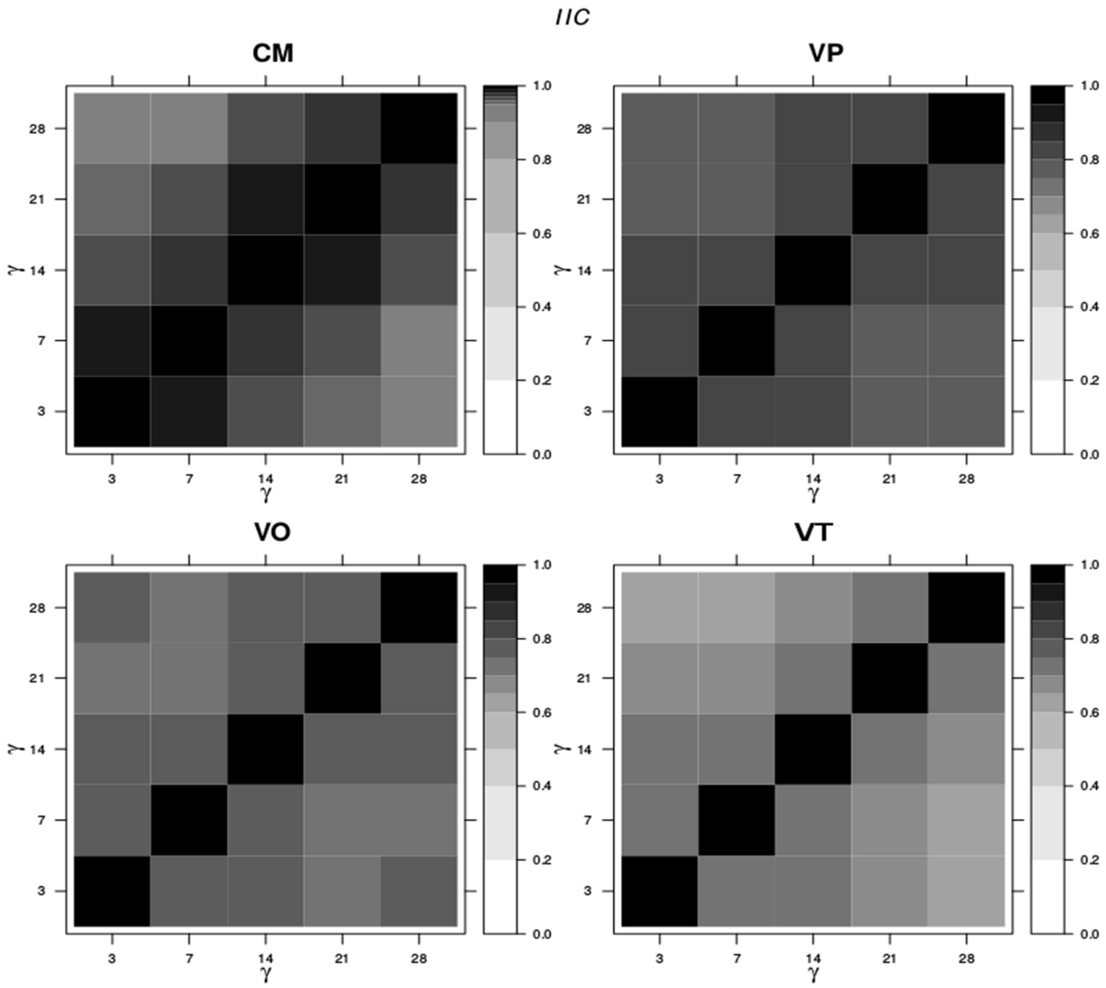

Figure S1.5.1: Kendall's τ correlations between in-infection chain (*IIC*) calculated by using five values of infectious period γ: 3, 7, 14, 21 and 28 days. The top and left panel shows correlations for cattle movements (CM) network, bottom and left panel for veterinary officers visits (VO) network, top and right panel for veterinary practitioners visits (VP) network, and bottom down panel for veterinarians total visits (VT) network. All p's were < 0.05. The colour-scale for CM network is different in order to increase contrast.

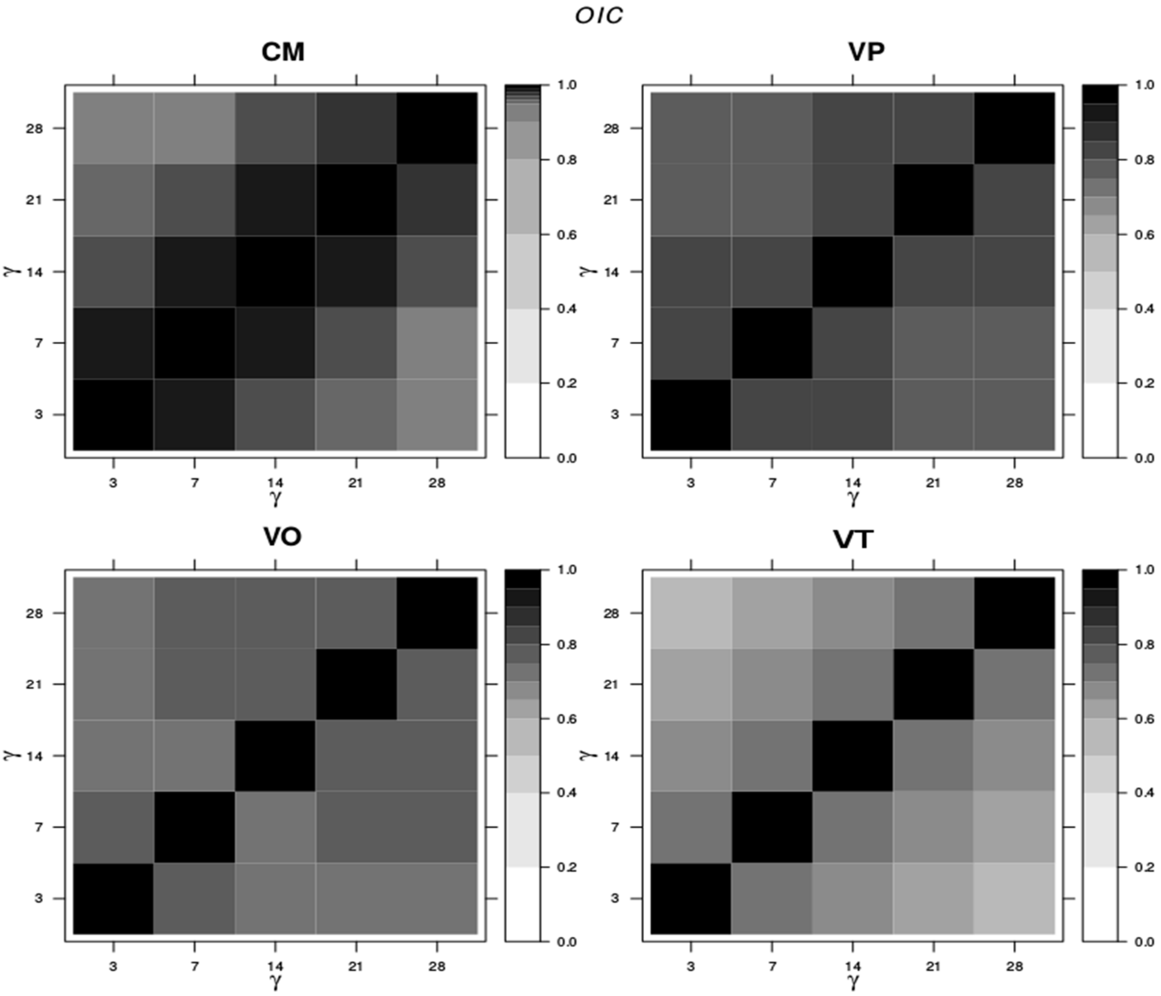

Figure S1.5.2: Kendall's τ correlations between out-infection chain (O*IC*) calculated by using five values of infectious period γ: 3, 7, 14, 21 and 28 days. The top and left panel shows correlations for cattle movements (CM) network, bottom and left panel for veterinary officers visits (VO) network, top and right panel for veterinary practitioners visits (VP) network, and bottom down panel for veterinarians total visits (VT) network. All p's were < 0.05. The colour-scale for CM network is different in order to increase contrast.

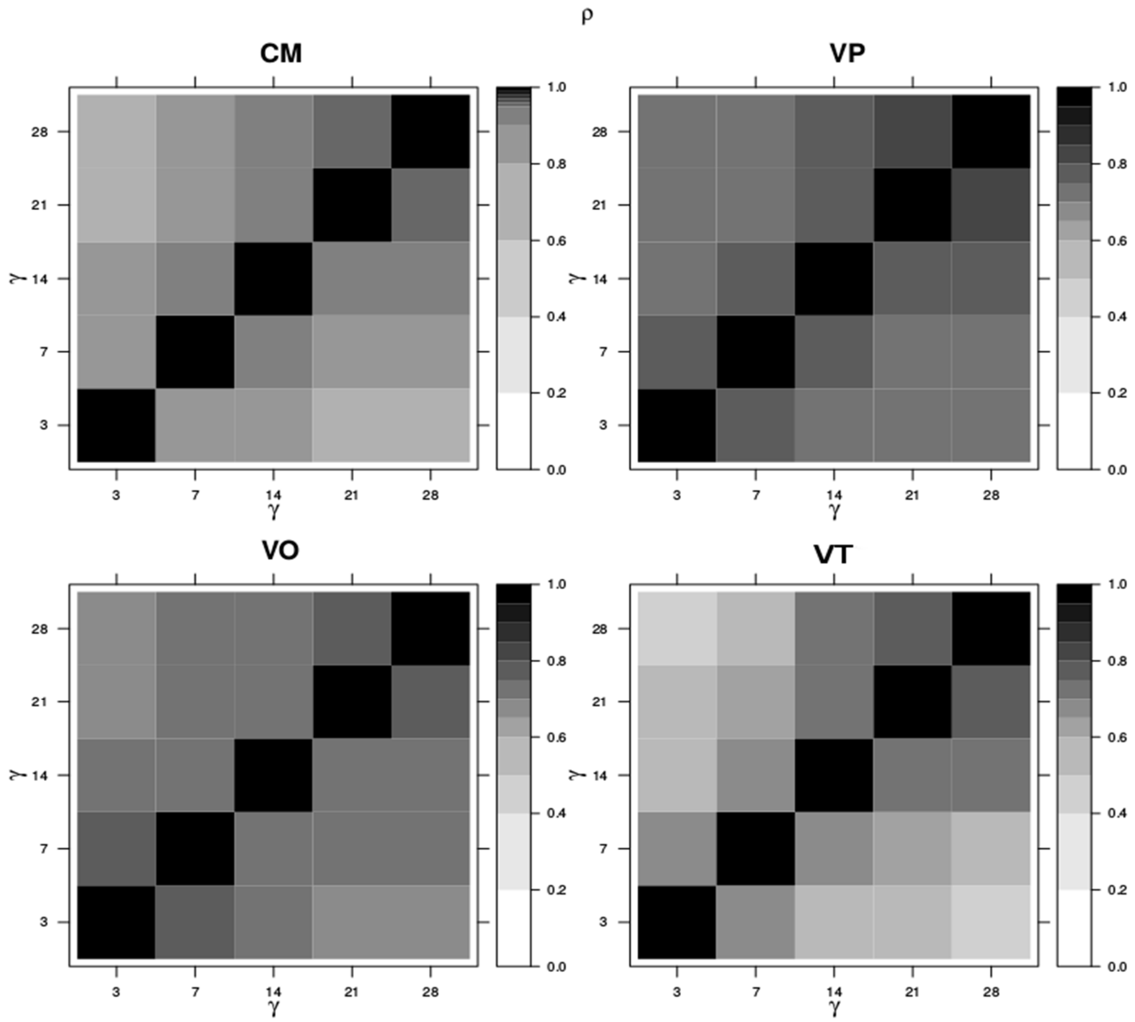

Figure S1.5.3: Kendall's τ correlations between infection potential (ρ) calculated by using five values of infectious period γ: 3, 7, 14, 21 and 28 days. The top and left panel shows correlations for cattle movements (CM) network, bottom and left panel for veterinary officers visits (VO) network, top and right panel for veterinary practitioners visits (VP) network, and bottom down panel for veterinarians total visits (VT) network. All p's were < 0.05. The colour-scale for CM network is different in order to increase contrast.

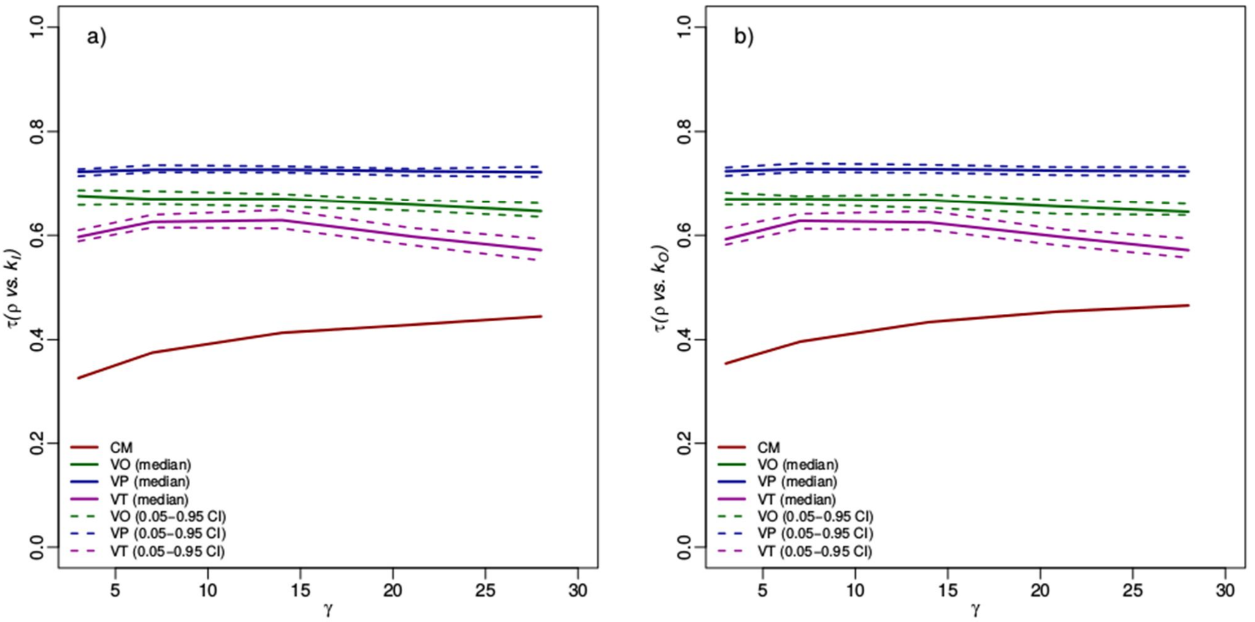

Figure S1.5.4: Kendall's τ correlations between: a) infection potential (ρ) and in-degree (k_I); c) infection potential (ρ) and out-degree (k_O). Correlations were calculated for increasing values of infectious period γ: 3, 7, 14, 21 and 28 days. The red lines represent the cattle movement (CM) networks; the green solid lines represent the median value for veterinary officer (VO) networks; the blue solid lines represent the median value for veterinary practitioner (VP) networks; and the purple solid lines represent the combined veterinarians total visits (VT) network. (Dashed lines represent the 5th and 95th percentile among the 20 simulations.

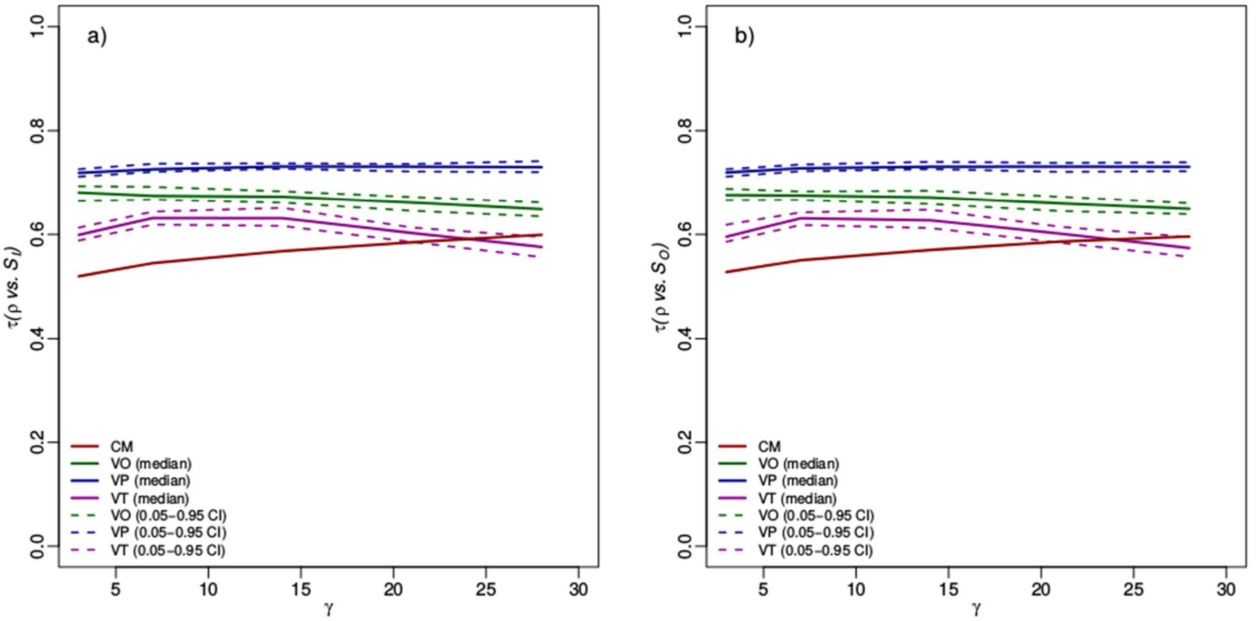

Figure S1.5.5: Kendall's τ correlations between: a) infection potential (*ρ*) and in-strength (S_I); b) infection potential (*ρ*) and out-strength (S_O). Correlations were calculated for increasing values of infectious period *γ*: 3, 7, 14, 21 and 28 days. The red line represents the cattle movement (CM) network; the green solid line represents the median value for veterinary officers (VO) network (dashed green lines the 5th and 95th percentile among the 20 simulations); the blue solid line represents the median value for veterinary practitioners (VP) network (dashed blues lines the 5th and 95th percentile among the 20 simulations); and the purple solid line represents the median value for veterinarians total visits (VT) network (dashed purple lines the 5th and 95th percentile among the 20 simulations).
