# Supplementary material for: The Potential Role of Direct and Indirect Contacts on Infection Spread in Dairy Farm Networks
Source: PLoS Comput Biol. 2017 Jan 26;13(1):e1005301. doi: 10.1371/journal.pcbi.1005301 (PMC5268397; doi:10.1371/journal.pcbi.1005301)
Supplement: S1 Data — This includes three CSV files reporting a configuration of the contacts list for the considered networks (cattle movement, veterinary officers, and veterinary practitioners). (ZIP) [file pcbi.1005301.s002.zip › SupData/Readme.docx]

Supporting dataset

TITLE:

The potential role of direct and indirect contacts on infection spread in dairy farm networks

AUTHORS:

Gianluigi Rossi, Giulio A. De Leo, Stefano Pongolini, Silvano Natalini, Luca Zarenghi, Matteo Ricchi, Luca Bolzoni

DATASET CONTENT:

edglist.cm.csv : list of cattle movement network edges for year 2013

edglist.vo.csv : list of veterinary officers network edges for year 2013 (h = 0)

edglist.vp.csv : list of veterinary practitioners network edges for year 2013 (h = 0)

Files contain:

source farm # (1 to 1349)

destination farm # (1 to 1349)

day (from 1, 2013-01-01, to 365. 2013-12-31)
